# Protection of Armadillo/β-Catenin by Armless, a Novel Positive Regulator of Wingless Signaling

**DOI:** 10.1371/journal.pbio.1001988

**Published:** 2014-11-04

**Authors:** Gerlinde Reim, Martina Hruzova, Sandra Goetze, Konrad Basler

**Affiliations:** Institute of Molecular Life Sciences, University of Zurich, Zurich, Switzerland; Stanford University School of Medicine, Howard Hughes Medical Institute, United States of America

## Abstract

The Wingless (Wg/Wnt) signaling pathway is essential for metazoan development, where it is central to tissue growth and cellular differentiation. Deregulated Wg pathway activation underlies severe developmental abnormalities, as well as carcinogenesis. Armadillo/β-Catenin plays a key role in the Wg transduction cascade; its cytoplasmic and nuclear levels directly determine the output activity of Wg signaling and are thus tightly controlled. In all current models, once Arm is targeted for degradation by the Arm/β-Catenin destruction complex, its fate is viewed as set. We identified a novel Wg/Wnt pathway component, Armless (Als), which is required for Wg target gene expression in a cell-autonomous manner. We found by genetic and biochemical analyses that Als functions downstream of the destruction complex, at the level of the SCF/Slimb/βTRCP E3 Ub ligase. In the absence of Als, Arm levels are severely reduced. We show by biochemical and in vivo studies that Als interacts directly with Ter94, an AAA ATPase known to associate with E3 ligases and to drive protein turnover. We suggest that Als antagonizes Ter94's positive effect on E3 ligase function and propose that Als promotes Wg signaling by rescuing Arm from proteolytic degradation, spotlighting an unexpected step where the Wg pathway signal is modulated.

## Abstract 2

This study uses an RNAi screen in *Drosophila* to identify a UBX protein, Armless, as a novel positive regulator of the important Wingless/Wnt signaling pathway, acting to stabilize Armadillo/?-Catenin by antagonizing its turnover.

## Author Summary

The Wg/Wnt signaling pathway, found in most animals, is essential for regulating tissue growth and the formation of different cell types during development. Defects in the Wg/Wnt signaling relay can have serious consequences, ranging from aberrant organ patterning to malignant tumor formation. A pivotal step in the transmission of the Wg/Wnt signal is the stabilization of the protein Armadillo/β-Catenin, a key component of the pathway. However, the means by which the levels of this protein are regulated remain unclear. Here, we describe a novel control point of Armadillo/β-Catenin levels. Using RNA interference, we performed a screen in the fruit fly *Drosophila melanogaster* and identified Armless, a protein whose biological function was previously unknown, as a novel regulator of Wg/Wnt signaling, essential for the Wg/Wnt-dependent expression of downstream target genes. Our experiments suggest that Armless interferes with the tagging of Armadillo/β-Catenin with ubiquitin, thereby sparing it from proteasomal degradation. We also show that Armless directly interacts in vivo with Ter94, a ubiquitous ATPase involved in protein turnover. Our results suggest that Armless antagonizes Ter94's function in protein turnover, thereby acting as a positive regulator of Wg/Wnt signaling by promoting the stabilization of Armadillo/β-Catenin.

## Introduction

The *wingless* (*wg*) gene was found nearly forty years ago with the characterization of a *Drosophila* mutant without wings [1]. The gene encodes a secreted glycoprotein, the founding member of the Wnt family of signaling proteins [2]. In the decades following its discovery, Wg/Wnt signaling has been shown to be essential during embryogenesis. Indeed, it is important throughout an organism's life, controlling also the homeostasis of different organs, for example, regeneration of epithelial cells in the intestine—the aberrant behavior of these cells in cancer is caused by constitutive Wg/Wnt signaling, which is consequently a key focus of medical and translational research [3],[4].

The relay of the Wg signal is controlled at different levels. However, the pivotal step is the regulation of the levels of Armadillo (Arm)/β-Catenin, the key transducer of the Wg/Wnt pathway. A multiprotein complex consisting of the scaffold proteins Axin and APC and the kinases Shaggy/GSK3β and Casein kinase I (CKI) recruits and phosphorylates Arm/β-Catenin. This marks Arm/β-Catenin for ubiquitination by the SCF/Slimb/βTRCP E3 ubiquitin ligase and subsequent degradation by the ubiquitin-proteasome system (UPS). When Wg/Wnt binds its receptors at the cell membrane, degradation of Arm/β-Catenin is prevented, presumably by protein interactions that lead to the dissociation of the E3 ubiquitin ligase from the Arm/β-Catenin destruction complex [5]. As a consequence, Arm/β-Catenin translocates into the nucleus, where it adopts its role as a transcriptional effector of Wg/Wnt signaling. Although this step is crucial, and is a potential point of regulation, little is known about the players involved in the processing of Arm/β-Catenin and its ultimate degradation.

In a genome-wide RNA interference (RNAi) screen we isolated Armless (Als) as a regulator of proximodistal growth of *Drosophila* limbs, and show in subsequent analyses that it exerts its function in the Wg pathway. Detailed genetic studies demonstrate that Als acts downstream of the destruction complex, at the level of the SCF/Slimb/βTRCP E3 Ub ligase. Cells depleted for Als exhibit strongly reduced Arm protein levels. Importantly, the activity of a constitutively active form of Arm, Arm^S10^, which cannot be phosphorylated and hence escapes ubiquitination and proteasomal degradation, is insensitive to depletion of Als. Using immunopurification and mass spectrometry analysis we found that Ter94 interacts with Als. Ter94 is an AAA ATPase associated with protein turnover and proteasomal degradation [6]. In sum, our data suggest that Als acts downstream of the Arm/β-Catenin destruction complex to positively regulate Arm protein levels, possibly by rescuing Arm from ubiquitination via Slimb. The human ortholog of Als, UBXN6, can substitute for Als in *Drosophila*, and Wnt target gene expression was impaired upon knock-down of UBXN6 in HEK-293 cells. We thus infer that Als and UBXN6 represent regulators of a conserved mechanism that ensures appropriate levels of Armadillo/β-Catenin by antagonizing its entry into the UPS.

## Results

### *CG5469/armless* Is Required for Wing Growth

**Figure 1:**
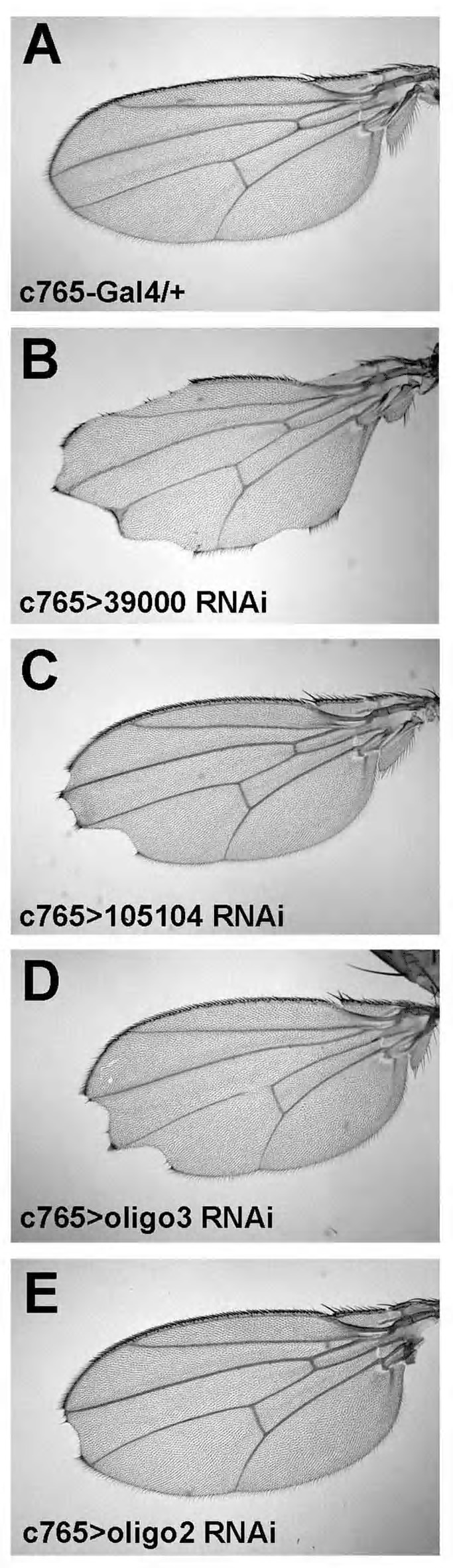
*als^RNAi^* causes notched wing margins. *c765-Gal4* drives the expression of different *UAS-als^RNAi^* transgenes in the entire wing primordium at 29°C, which results in nicked wing margins, accompanied by loss of sensory bristles. (A) *Gal4* driver only, (B) VDRC line 39000, (C) VDRC line 105104, (D) oligo3, (E) oligo2.

Among its numerous developmental roles, Wg controls growth and patterning of the *Drosophila* wing. A hallmark phenotype of reduced *wg* function is nicked wings with reduced size. Positively acting components of the Wg pathway mostly display similar phenotypes when depleted via RNAi during larval stages. We established and used a tester system that allowed screening for genes involved in wing growth and margin formation. Our screen covered approximately 83% of all the predicted 14,306 genes of *Drosophila*. The details of the screen will be published elsewhere. *CG5469* behaved as expected of a putative Wg signaling component: RNAi targeting its transcripts affected wing size in a non-allometric manner and caused a notched margin and loss of sensory bristles. This phenotype could be reproduced by the expression of nine independent *UAS-CG5469^RNAi^* lines with different Gal4 drivers (Figures 1B–1E and S1A–S1J). Expression of *CG5469^RNAi^* effectively reduced mRNA levels of *CG5469* (Figure S1L, cf. Figure 1B–1E), and haplo-deficiency exacerbated the wing phenotypes (Figure S1M and S1N); these phenotypes were rescued upon overexpression of *CG5469*, as detailed in Text S1 and shown in Figure S2.

Wing notches could be caused by increased apoptosis or reduced cell proliferation. To discriminate between these possibilities, we experimentally accelerated cell divisions by *string/cdc25* overexpression [7], and inhibited apoptosis by Diap1 overexpression. Only *string/cdc25* overexpression restored marginal wing tissue loss caused by depletion of *CG5469* (Figure S3F). This finding is consistent with a role of *CG5469* in Wg signaling, as impaired Wg pathway activity affects primarily proliferation (Figure S3A–S3C; [8],[9]). Because of its mutant phenotypes, which are caused by severely reduced levels of Arm (see below), we refer to *CG5469* as *armless (als*; for the nomenclature see also Text S1).

### *als* Interacts with the Wg Pathway and Is Required for Wg Target Gene Expression

To further test whether *als* interacts with Wg signaling, we analyzed the effects of *als^RNAi^* in different, sensitized backgrounds. Interfering with the Wg pathway at various levels leads to typical wing margin notches and loss of distal wing tissue (Figure S3G–S3J). These phenotypes were enhanced upon *als* depletion (Figure S3G′–S3J′). To test whether *als* is also involved in the Wg pathway in other developmental contexts, we expressed *als^RNAi^* in the primordia of the dorsal thorax and the legs. Again, consistent with a role of *als* in the Wg pathway (Figure S4D and S4H; [10],[11]), thorax and scutellum sizes were affected (Figure S4B, S4C, S4F, and S4G, cf. Figure S4A), and showed reduced or missing microchaete, a slight misorientation of remnant micro- and macrochaete, and mild thoracic clefts (Figure S4B and S4C). Depletion of *als* in the leg discs caused a reduction of tarsal segments along the proximodistal axis and a dorsolateral shift of the sex combs—phenotypes characteristic of reduced Wg signaling (Figure S4J–S4M; [12],[13]). Stronger *als^RNAi^* expression caused a dorsalization of the leg as judged from the mirror-image patterning of leg trichomes (Figure S4K and S4M). To analyze the phenotype at earlier stages of development, we expressed *als^RNAi^* or Als^HA^ (a dominant-negative-like version; see Text S1) in the embryo with *da-Gal4*. We found that cuticles were shorter than in control embryos, with ventral denticle belts partially fused, which is indicative of a segment polarity defect (Figure S4S–S4V, S4X, and S4Y). These phenotypes are reminiscent of milder Wg loss-of-function phenotypes caused by *arm^RNAi^* or *UAS-Lgs^17E^* expression (Figure S4P–S4R and S4W) by *da-Gal4*.

**Figure 2:**
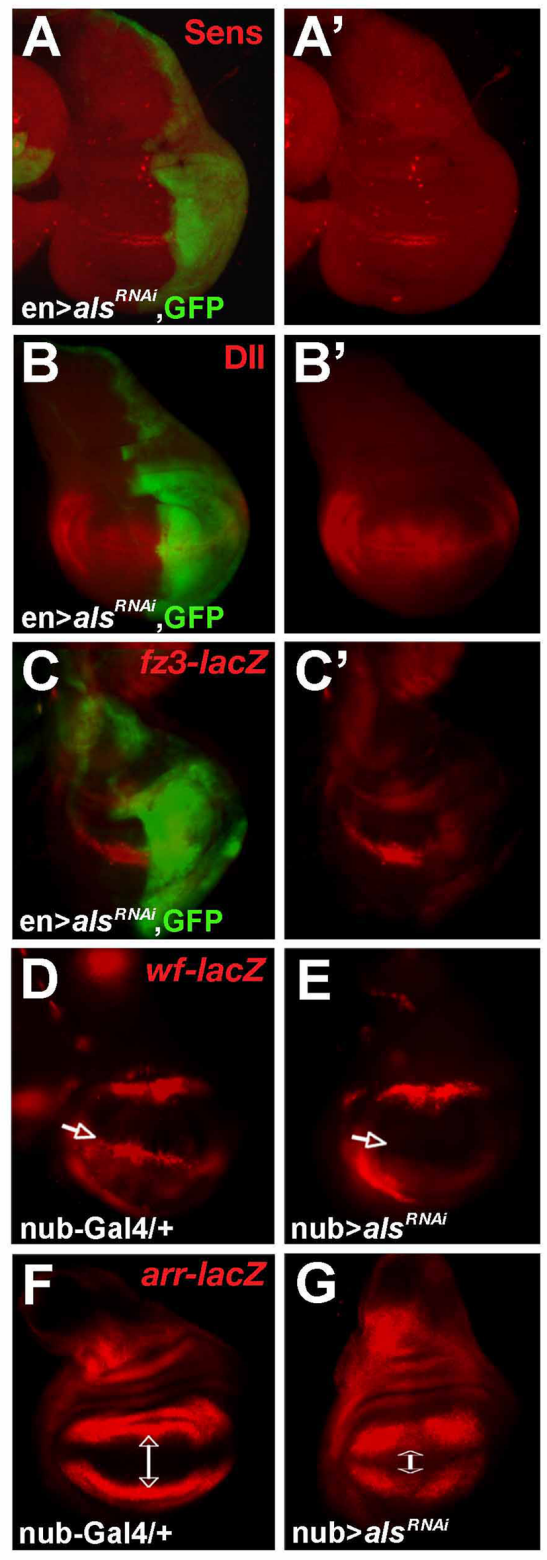
*als* is required for Wg-dependent target expression. L3 wing imaginal discs; anterior is to the left, posterior is to the right. *als^RNAi^* expression in the P-compartment of wing imaginal discs (visualized by the co-expression of *UAS-GFP*, green fluorescence) (A–C′) or in the entire wing pouch (D–G) causes loss of expression of Wg-signaling-dependent target genes—Senseless (A and A′) (line 39000), Distalless (B and B′) (line oligo3), *frizzled3* (C and C′) (line oligo3), or *wingful* (D and E; white arrows indicate the Wg-dependent expression domain of *wf* at the D/V border which is lost in E.) (line 39000)—and ectopic expression of *arrow*, which is a negative target (G c.f. F; white arrows indicate the extent of the domain that is free from *arrow-lacZ* expression) (line oligo3).

To further test whether *als* is required for Wg signaling output, we monitored the expression of well-known Wg target genes. Wg is secreted from a stripe of cells at the dorsoventral (D/V) border and triggers a transcriptional response in cells along the D/V axis of imaginal wing discs (the future proximodistal axis of the adult wing). Wg activates the proneural gene *senseless (sens)* in sensory organ precursors, which develop into the bristles of the adult wing margin [8],[14]. *als^RNAi^* expression in the P-compartment resulted in a loss of Sens therein (Figure 2A and 2A′). Expression of Distalless (Dll), *frizzled3* (*fz3*), and *wingful (wf)*, which are activated by Wg in a broader region centered on the D/V border [15],[16], was strongly reduced or lost in response to reduction of *als* function (Figure 2B–2E). Conversely, *arrow (arr)*, which is normally repressed by Wg signaling in central pouch regions (Figure 2F; [17]), became ectopically activated towards the central wing pouch (Figure 2G). In sum, these observations strongly suggest that *als* encodes a component with a positive role in the Wg pathway.

To try to gain insight into the pathways *als* might regulate, we further looked at *als* expression. In situ hybridization and *lacZ* reporter transgenes indicated that *als* is expressed at early third instar wing imaginal discs at the center of the pouch adjacent to the D/V border and, at faint levels, throughout the wing primordium (Figure S5A–S5C). Furthermore, the expression of *UAS*-*als^RNAi^* in the P-compartment of wing imaginal discs caused a strong decrease in *als* expression (Figure S5B, inset). Interestingly, although normal when Wg signaling is inhibited, *als* expression could be upregulated when Wg signaling was activated by the overexpression of either Wg or Arm^S10^, and stimulation of Wg signaling by the inhibition of GSK3β caused elevated Als and *als* levels (Figures S5D and 8A). This is consistent with *als* functioning in the Wg pathway and having its activity fine-tuned by feedback mechanisms that ensure maximum pathway activity.

### *als* Is Not Required for Notch or Hedgehog Signaling

Wing notching is a phenotype characteristic not only of impaired Wg signaling, but also of reduced Notch signaling. The activities of both pathways are required to establish the D/V border and subsequent proximodistal wing growth. To determine whether *als* also plays a role in the Notch pathway, we analyzed the expression of the following Notch targets: (1) the proneural gene *e(spl)m8* (Figure S6A; [18]), (2) a Notch responsive element (NRE) reporter (Figure S6A inset; [19]), and (3) the *vestigial (vg)* “boundary enhancer” (Figure S6B; [20]). In contrast to what we observed for the Wg targets above, the expression patterns of these Notch reporters were unaltered upon *als* depletion (Figure S6A′–S6B″). Moreover, neither did reduction of *als* function suppress the phenotypes caused by elevated Notch signaling (Figure S6D and S6F, cf. Figure S6C and S6E), nor did it ever mimic a neurogenic thorax phenotype or fused tarsal segments (Figure S4), which are both typical for reduced Notch signaling. Finally, we monitored the expression of the *wg* gene itself, which is under control of Notch activity [21]–[23]. Neither *wg* transcription nor Wg protein expression were affected at the D/V boundary upon *als^RNAi^* expression (Figure S7A–S7G).

We also tested other pathways involved in wing growth and patterning, such as Hedgehog (Hh) signaling. Increased Hh signaling causes wing overgrowth predominantly along the anteroposterior (A/P) axis, along with ectopic veins in the A-compartment (Figure S6G). *als* depletion impaired wing growth along the proximodistal axis, which is under control of Wg signaling, but not along the A/P axis, and it did not alter the vein patterning defects caused by increased Hh signaling (Figure S6H). Furthermore, expression of the Hh target gene *patched (ptc)* was not affected by *als* depletion (Figures S6I, S6J, and 8E). In addition, we tested Upd/Jak/Stat signaling and EGFR/Ras signaling and found that none of these pathways was affected by *als* depletion (Figure S6K–S6N). Our findings therefore suggest a specific role for *als* in Wg signaling in the tissues that we have tested.

### Mapping Als Action by Cell Autonomy and Epistasis Analyses

**Figure 3:**
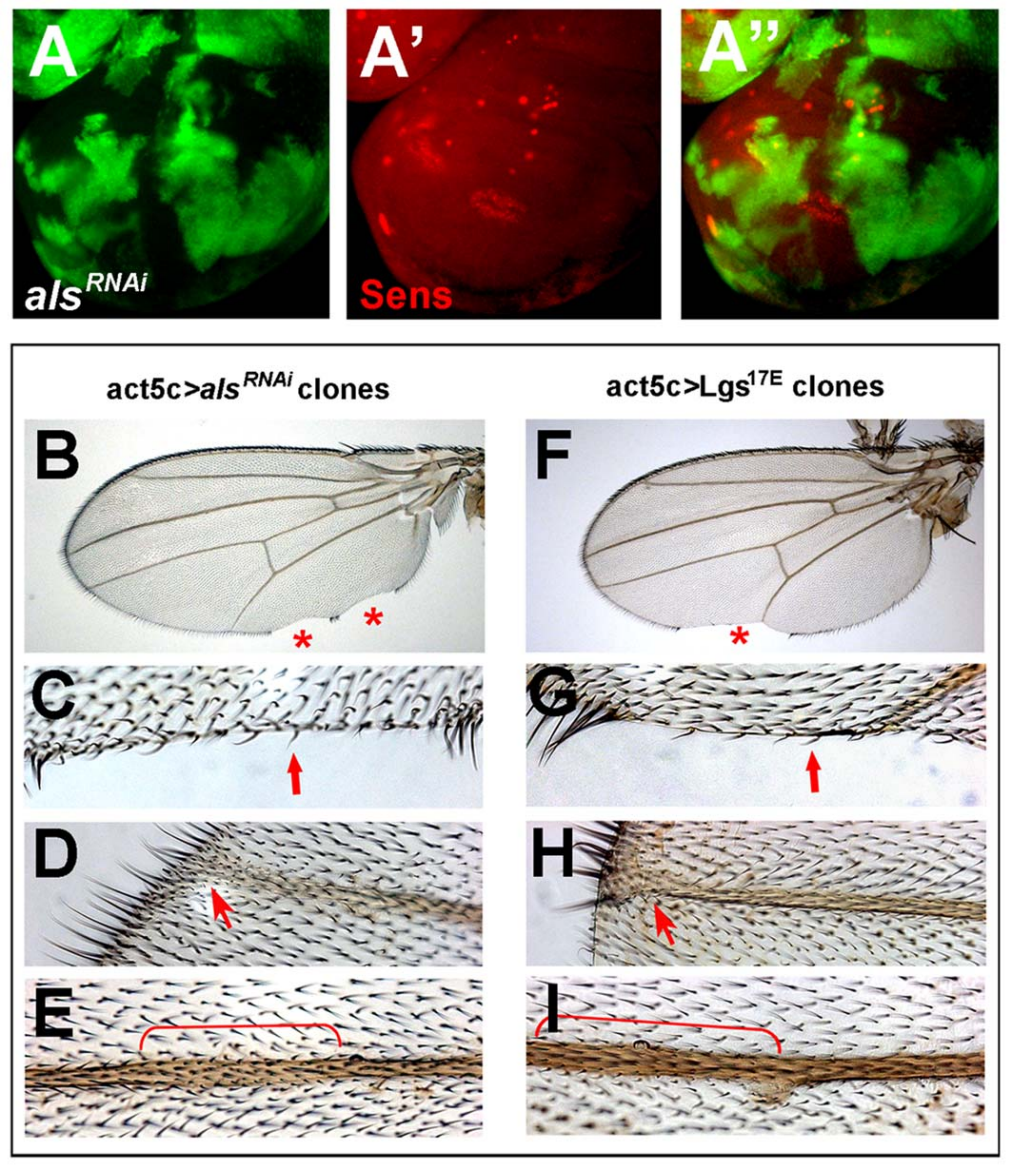
*als* acts in a cell-autonomous manner. Wg target gene expression (Sens) is affected cell-autonomously upon impaired *als* function as judged from wing disc analysis at the late L3 stage (A–A″). GFP expression indicates cell clones expressing *als^RNAi^* line *oligo3^10UAS^* (A and A″). Adult wing phenotypes coincide with cell clones that experienced *als* depletion, as marked by *forked* wing hairs (B–E). This is reminiscent of adult wing phenotypes obtained when Wg signaling is impaired (Lgs^17E^ expression) (F–I). Red asterisks (B and F) indicate notches at the wing margin; red arrows (C and G) indicate wing margin notches accompanied by loss of mechano- and sensory bristles; red arrows (D and H) and brackets (E and I) indicate broadened veins.

To investigate whether Als functions in Wg producing or receiving cells, *UAS*-*als^RNAi^* was expressed in clones of wing imaginal cells marked by GFP co-expression. Expression of the Wg target Sens was lost in early induced clones (Figure 3A–3A″). This effect is cell-autonomous, i.e., it also occurs when mutant cells are close to wild-type cells that produce Wg. Adult wing clones, marked by *UAS*-*forked^RNAi^* co-expression, had wing notches and thickened veins (Figure 3B–3E), both of which were tightly associated with mutant cells. We observed similar phenotypes when Wg signaling was cell-autonomously impaired (Figure 3F–3I).

**Figure 4:**
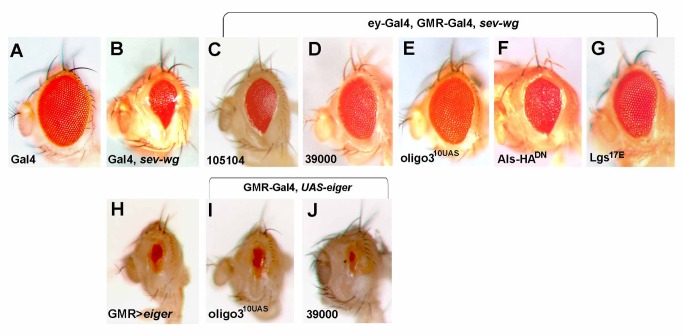
*als* acts downstream of Wg. Control eye with *ey-Gal4, GMR-Gal4/+* (A). The small eye phenotype based on *sev-wg* expression (B) can be suppressed by the expression of different *UAS*-*als^RNAi^* transgenes (C–E), by expression of *UAS*-*als^HA(DN)^* (F), and by overexpression of Lgs^17E^, which suppresses Wg signaling (G). The small eye phenotype based on enhanced apoptosis (H) is not altered upon *als* depletion (I and J).

To map where Als acts in the Wg pathway, we used phenotypic assays based on the activation or inactivation of Wg signaling in the eye or the wing. Expression of Wg under control of the *sevenless (sev)* enhancer causes small, narrow eyes that lack interommatidial bristles (Figure 4B; [24],[25]). This phenotype is partially suppressed when Wg signal transduction is hindered, for example, by the expression of a dominant-negative form of Lgs (Lgs^17E^ [26]; Figure 4G). Impaired *als* function also causes a suppression of the *sev-wg* phenotype (Figure 4C–4F). In contrast, depletion of *als* did not alter the small eye phenotype caused by the overexpression of Eiger (Figure 4H–4J), a trigger of the apoptotic pathway [27].

**Figure 5:**
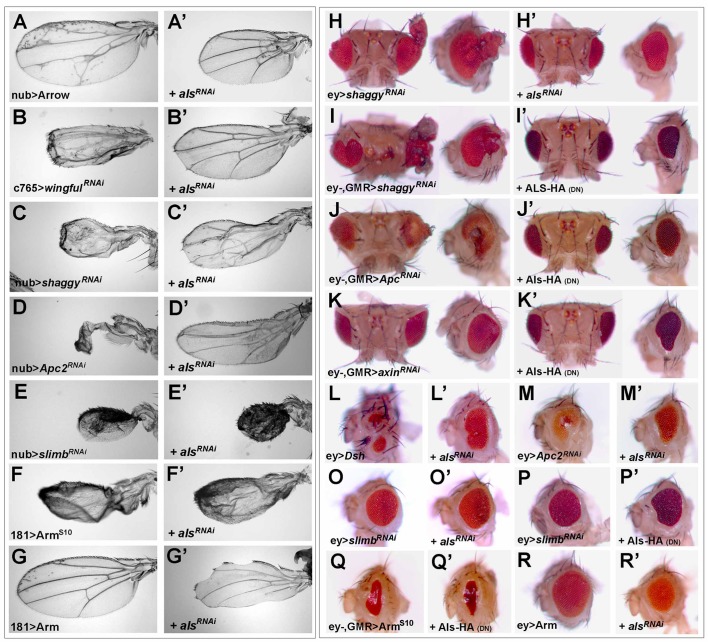
*als* functions downstream of the Arm/β-Catenin destruction complex and upstream of activated Armadillo. Overexpression of the positive components Arrow (A), Dishevelled (L), Arm^S10^ (F and Q), or Arm (G and R) and depletion of negative components such as *wingful* (B), *shaggy* (C, H, and I), *apc* (D and J), *slimb* (E, O, and P), and *axin* (K) cause phenotypes that reflect ectopic Wg signaling: ectopic sensory bristles (A–G), ectopic veins (A–C), and tissue overgrowth (A, H–K, O, and P), but also reduced organ size upon strong pathway activation (B–F, L, M, and Q) and caused ectopic head cuticle (H–J, L, and M). Co-expression of *als^RNAi^* could suppress these phenotypes in the wing (A′–D′) and the eye (H′–M′). Phenotypes based on *slimb^RNAi^* and Arm^S10^ expression could not be suppressed upon *als* depletion (E′, O′, P′, F′, and Q′). Arm overexpession could not rescue *als^RNAi^* phenotypes (G′ and R′). Heads were photographed from dorsal views (left pictures of H–K′) or lateral views, anterior to the left (right pictures of H–K′, L–R′). *als^RNAi^* lines: *oligo3^10UAS^* (A′–E′) and for the eye analysis; *oligo2_5′utr* (G′).

We manipulated the Wg signaling cascade in the wing and the eye at successively more downstream positions and assessed the requirement of Als for the manifestation of the respective Wg gain-of-signaling phenotypes (see legend of Figure 5 for a detailed description of phenotypes). First, we found that Als depletion suppressed Wg pathway activation caused by RNAi of *notum/wingful* (encoding an extracellular inhibitor of Wg [28],[29]) or by overexpression of Arrow (co-receptor of Wg, [17]) or Dishevelled (Figure 5A–5B′, 5L, and 5L′). Next, we tested knock-downs of components of the destruction complex, both in the wing and the eye, and found that aberrant Wg output was suppressed by *als^RNAi^* for Axin, APC, and Shaggy/GSK3β (Figure 5C, 5C′, 5D, 5D′, 5H–5K′, 5M, and 5M′). Only the phenotypes of RNAi against the SCF/Slimb/βTRCP E3 ubiquitin ligase [30] and expression of Arm^S10^, a constitutively active form of Arm/β-Catenin [31], could not be ameliorated by *als* depletion (Figure 5E–5F′ and 5O–5Q′). Interestingly, wild-type Arm could not overcome the *als^RNAi^* phenotypes in the wing and the eye, and did not lead to the emergence of ectopic sensory bristles (Figure 5G, 5G′, 5R, and 5R′). We interpret these genetic findings to indicate that Als functions upstream of Arm, but downstream of the Arm/β-Catenin destruction complex, possibly at the level of the SCF/Slimb/βTRCP E3 Ub ligase.

### *als* Encodes a UBX Domain Protein That Localizes in the Cell Cortex and Depends on a Functional PUG-Domain

Three different protein domains can be distinguished within Als: (1) an N-terminal coiled-coil domain, (2) a PUG domain, and (3) a UBX domain (Figure S9). Coiled-coil domains function in protein oligomerization, whereas the PUG und UBX domains are found in ubiquitin regulatory proteins [32]. To find out more about its molecular role, we tested where Als localizes within cells and which of its protein domains are functionally required. N-terminally tagged ^HA^Als localized close to the cell membrane in a punctate ring pattern (Figure S8A–S8B′), with strongest expression in the apical region of the cell (Figure S8C, S8D, S8E). This localization is reminiscent of that of E-Cadherin, Arm, and the Wg receptor Frizzled2 (Fz2, Figure S8C–S8E″). We then expressed Als protein variants that lacked particular domains and assessed whether these could rescue the *als^RNAi^* phenotypes. For this we created tagged and untagged constructs that were either sensitive or insensitive to the RNAi used. Expression of Als^ΔN^ and Als^ΔPUG^ did not alter the *als^RNAi^* phenotype (Figure S9A–S9I), irrespective of their tag or targetability. However, expression of Als^ΔUBX^ as well as full-length Als protein substantially rescued the wing phenotypes caused by *als^RNAi^* (Figure S9J–S9L). This suggests that the UBX domain is dispensable for Als function, whereas the N-terminal and the PUG domains are both important. The requirement of the PUG domain implicates Als in playing a role in a ubiquitin-mediated protein degradation process.

### Als Interacts with Ter94, a Conserved ATPase Involved in Protein Turnover

**Figure 6:**
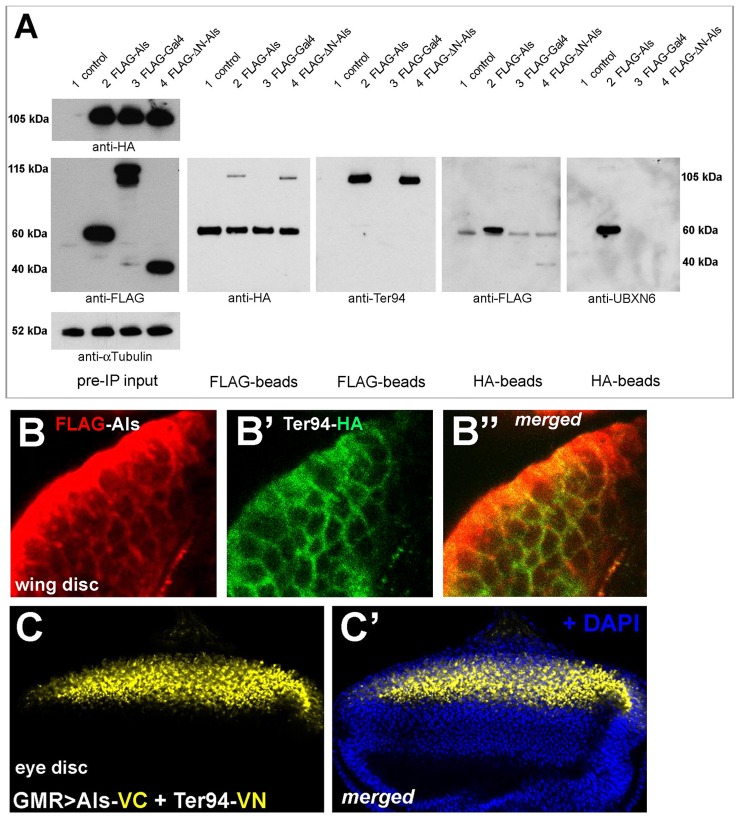
Als co-localizes and interacts with Ter94. (A) Western blot analysis after immunoprecipitation (IP) of ^FLAG^Als or Ter94^HA^. Condition 1: negative control (empty *pUAST-attB* vector), condition 2: ^FLAG^Als (60 kDa), condition 3: negative control (^FLAG^Gal4, 115 kDa), condition 4: ^FLAG^ΔN-Als (40 kDa); Ter94^HA^ (105 kDa) was co-expressed in conditions 2–4. Ter94 binds to full-length Als (condition 2) and ΔN-Als (condition 4), and vice versa. The anti-human-UBXN6 peptide antibody recognizes only the full-length Als, but not ΔN-Als. (B–B″) Transgene expression by *nubbin*-*Gal4* in wing imaginal disc cells shows that ^FLAG^Als co-localizes with Ter94^HA^. (C and C′) Co-expression of Als-VC and Ter94-VN with *GMR-Gal4* in eye imaginal discs results in a Venus YFP fluorescence signal, which demonstrates a physical interaction between Als and Ter94.

To further our understanding of Als function, we expressed HA-tagged variants of Als in *Drosophila* Kc-167 cells, and after affinity purification we subjected the samples to shotgun liquid chromatography–tandem mass spectrometry (LC-MS/MS). In three independent experiments using Als^HA^, ^HA^Als, and ^HA^Als^ΔUBX^, but not in the negative control sample, we identified Ter94 as the highest ranking protein interactor of Als (see Text S1), irrespective of the stimulation of the Wg pathway by Wg. Ter94 is the *Drosophila* ortholog of p97, an AAA ATPase found to be associated with ubiquitin-dependent protein degradation processes, and that can interact with different UBX domain proteins [33],[34]. To further validate the interaction between Als and Ter94 we co-expressed Ter94^HA^ together with ^FLAG^Als, ^FLAG^Als^ΔN^, and ^FLAG^Gal4, which served as a negative control, in Kc-167 cells. Western blot analyses following reciprocal immunoprecipitations showed that (1) Ter94^HA^ and endogenous Ter94 co-immunoprecipitated with ^FLAG^Als and ^FLAG^Als^ΔN^, and (2) ^FLAG^Als, ^FLAG^Als^ΔN^, and endogenous Als co-immunoprecipitated with Ter94^HA^ (Figure 6A). When we expressed Ter94^HA^ in wing imaginal discs, we found a strikingly similar sub-cellular distribution and co-localization with ^FLAG^Als (Figure 6B–6B″). Importantly, we demonstrated a physical interaction between Als and Ter94 in vivo with bimolecular fluorescence complementation (BiFC) analysis (Figure 6C and 6C′; [35]; see also Materials and Methods), where the co-overexpression of Als-VC and Ter94-VN resulted in a Venus-YFP fluorescent signal. In sum, our biochemical and genetic results indicate that Als molecularly interacts with Ter94. The UBX domain is dispensable for Als to bind to Ter94, which is in agreement with our finding that Als lacking the UBX domain could still rescue the *als^RNAi^* phenotype.

### Armadillo Levels Depend on *als* Activity

**Figure 7:**
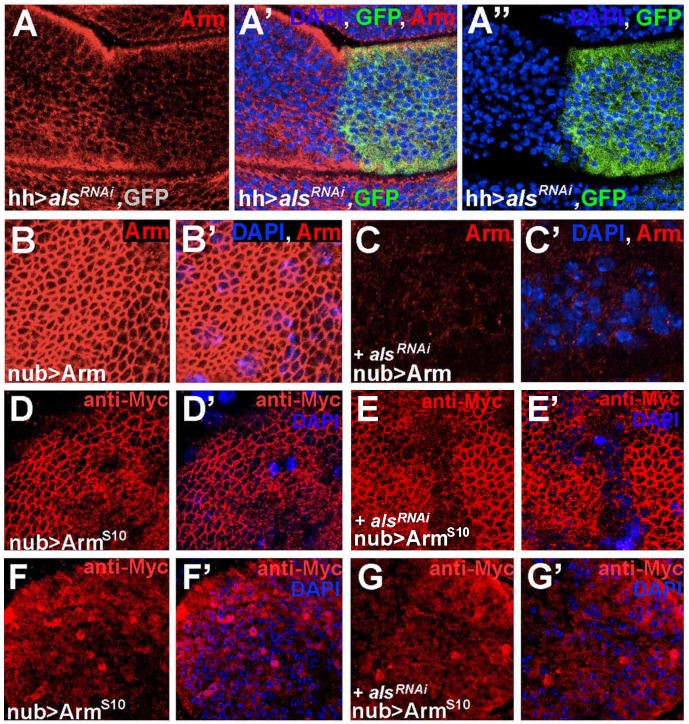
Reduction of Armadillo levels upon *als* depletion. (A–A″) *als* depletion (line *oligo3^10UAS^*) in the P-compartment of wing imaginal discs caused reduced Arm protein levels compared to the A-compartment (A′ and A″). CD8-GFP expression marks the cell membranes of the P-compartment, and DAPI staining marks all cellular nuclei. (B and B′) The expression of Arm driven from a transgene by *nub*-*Gal4* is similar to endogenous Arm (cf. Figure S8D′). (C and C′) Upon *als* depletion (line 39000), Arm levels were strongly reduced. (D–G′) DAPI staining (D′–G′) visualizes the large nuclei of the peripodial membrane (blue, out of the focal plane), which overlies the apical part of wing disc cells including Arm (in the focal plane of D–E′). The expression of Arm^S10-Myc^ (F–G′) was not altered upon depletion of *als* (line *oligo3^10UAS^*). (D–E′): apical confocal sections; (F–G′): deeper confocal *Z*-sections, which show enhanced cytoplasmic Arm^S10^ levels.

Our biochemical and genetic studies suggest that Als is functionally linked to the proteasomal degradation pathway. Since Als is positively required for Wg signaling, it could exert its function in the pathway either by suppressing the degradation of a positive component, or by enhancing the degradation of a negative component. We first analyzed the protein levels of key negative pathway components in wing imaginal discs. We expressed different *als^RNAi^* lines in the wing primordium with *nub-Gal4*; however, neither Axin nor Shaggy nor Apc2 levels were affected upon *als* depletion (Figure S10A–S10L); [36]). In contrast, we found that the protein levels of Arm, the key positive signaling component, were dependent on *als*: when *als^RNAi^* was expressed in the P-compartment under the control of *hh*-*Gal4*, Arm levels were strongly reduced in P but not A cells (Figure 7A–7A″). Even when we overexpressed Arm under the control of *nub*-*Gal4*, co-expression of *als^RNAi^* led to strongly reduced Arm levels (Figure 7C and 7C′, cf. Figure 7B and 7B′). DAPI staining shows that all cells have normally shaped nuclei and are thus not apoptotic (Figure 7A′, 7A″, 7C, and 7C′). Constitutively active, non-degradable Arm^S10^ localizes like Arm to the apical membrane upon overexpression (Figure 7D and 7D′), and was also observed at higher levels in the cytoplasm (Figure 7F and 7F′). Importantly, we found that Arm^S10^ levels were not altered in *als*-depleted wing imaginal disc cells (Figure 7E, 7E′, 7G, and 7G′). This is consistent with our finding that Arm^S10^ overexpression could completely overcome the phenotypes caused by *als* depletion (Figure 5F, 5F′, 5Q, and 5Q′). Other positively acting components of the Wg pathway, Arrow and Fz2, did not exhibit alterations in protein levels upon *als^RNAi^* expression (Figure S10M–S10P). The observation that Arm is subject to degradation in the absence of *als* function is in agreement with our observation that overexpressed Arm—in contrast to Arm^S10^—is not able to rescue *als^RNAi^* phenotypes in wing and eye primordia (Figure 5G′ and 5R′).

The rescue experiments with Arm and Arm^S10^ suggested that Als acts upstream of Arm's proteasomal degradation. To further corroborate this idea, we reduced the levels of *ubiquitin* in addition to *als* depletion. This led to a suppression of the *als^RNAi^* phenotypes in the wing and the eye (Figure S11I, S11L, and S11O, cf. Figure S11G, S11J, and S11M). Reduction of *ubiquitin* also suppressed Wg loss-of-function phenotypes that were based on elevated activity of the destruction complex by Axin overexpression (Figure S11A and S11C). In contrast, depletion of *ubiquitin* did not suppress the Lgs^17E^ overexpression phenotype, which intersects the Wg pathway downstream of the proteasome (Figure S11D and S11F). These findings support the idea that Als acts upstream of Arm's proteasomal degradation.

To check whether Als directly interacts with Arm, we carried out selected reaction monitoring, a highly sensitive mass spectrometry approach that allows the detection of low amounts of peptides in a complex sample [37]. Als and Ter94 were used as bait. In none of the studies was an interaction between Arm and Als detected. Additionally, in shotgun affinity purification mass spectrometry using Arm or Arm^S10^ as a bait we did not find Als, whereas, as expected, components of the degradation complex such as Apc2, Axin, Sgg, and CKI were detected in the cytoplasm (Text S1).

**Figure 8:**
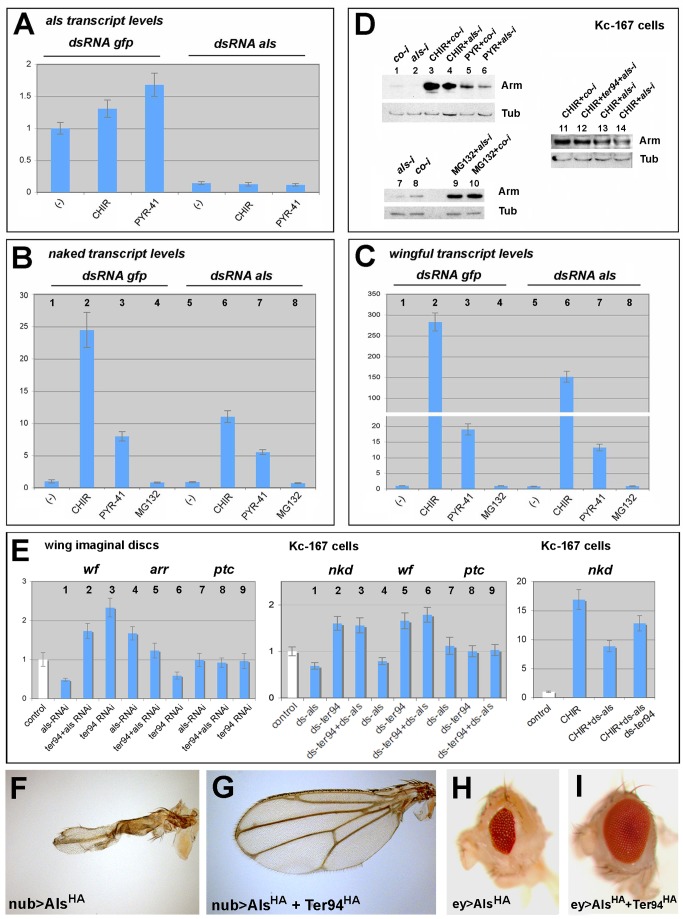
Als acts downstream of GSK3β and upstream of SCF/Slimb/βTRCP E3 Ub ligase. (A) *als* mRNA levels are downregulated upon *als*-dsRNA treatment. Stimulation of Wg signaling by inhibition of GSK3β (CHIR) or E1 ligase (PYR-41) causes an upregulation of *als* expression. (B and C) The positive Wg targets *nkd* and *wf* were induced upon GSK3β inhibition (bar 2 in [B and C]) and E1 ligase inhibition (bar 3 in [B and C]) in control cells. In *als*-dsRNA-treated cells, *nkd* and *wf* were strongly reduced in GSK3β-inhibited cells (bar 6 in [B and C]) and only mildly reduced in PYR-41-treated cells (bar 7 in [B and C]). Inhibition of the proteasome (MG-132) did not cause upregulated Wg target expression (bars 4 and 8). (D) Inhibition of GSK3β (CHIR) or E1 ligase (PYR) caused an increase of Arm protein levels in control cells (lanes 3 and 5); this increase was reduced upon *als*-dsRNA treatment (lanes 4 and 6); proteasome inhibition (MG132) caused elevated Arm levels in both control and *als*-dsRNA conditions (lanes 9 and 10). Combined depletion of *ter94* and *als* could ameliorate Arm levels (lane 12, cf. lanes 13 and 14, which represent *als* depletion based on different *als* siRNA target regions). (E) Control expression levels are depicted as white bars. In wing imaginal discs (left bar diagram), *als* depletion caused a strong reduction of *wf* expression, a positive Wg target gene (lane 1), and an elevation of *arr* expression, a negative Wg target gene (lane 4). Combined *als* and *ter94* depletion caused an elevation of *wf* (lane 2) or a decrease of *arr* (lane 5). No alteration was observed for the Hh target gene *ptc* (lanes 7–9). In Kc-167 cells (middle bar diagram), a co-depletion of *als* and *ter94* caused an upregulation of the positive Wg targets *nkd* and *wf* (lanes 3 and 6), which were reduced upon *als* depletion (lanes 1 and 4). No alteration was observed for the Hh target gene *ptc* (lanes 7–9). Reduced *nkd* expression of *als*-depleted, GSK3β-stimulated Kc-167 cells (right bar diagram) could be ameliorated upon co-depletion of *ter94*. (F–I) The wing and eye phenotypes caused by the expression of the dominant-negative-like Als^HA^ were suppressed by the overexpression of Ter94^HA^. co-i, control siRNA.

We further analyzed the consequences of *als* depletion for Wg signaling in Kc-167 cells (Figure 8). Stimulation of the Wg pathway by the inhibition of GSK3β led to strong transcriptional upregulation of the Wg targets *naked (nkd)* and *wingful (wf)* (Figure 8B and 8C, bar 2), and an increase in Arm levels (Figure 8D, lanes 3 and 11). Consistent with the in vivo data, double-stranded RNA (dsRNA)–mediated knockdown of *als* (Figure 8A) caused a reduction in *nkd* and *wf* expression in stimulated cells (Figure 8B and 8C, bar 6, cf. bar 2). Importantly, the increase in Arm levels based on GSK3β inhibition was significantly reduced when *als* was knocked down (Figure 8D, lane 4, cf. lane 3, and lane 13 and 14, cf. lane 11). When the pathway was stimulated by inhibiting E1 Ub ligase activity, which caused a milder upregulation of *nkd* and *wf* than the GSK3β inhibition (Figure 8B and 8C, bar 3; see also Materials and Methods), transcriptional upregulation was still decreased upon *als* depletion (Figure 8B and 8C, bar 7), but to a far lesser extent; at the same time, Arm was reduced upon *als* depletion (Figure 8D, lane 6, cf. lane 5). Inhibition of the 26S proteasome increased Arm levels in both control and *als*-dsRNA conditions (Figure 8D, lanes 9 and 10).

When we depleted *ter94* in wing imaginal discs and in Kc-167 cells, we found an upregulation of the positive Wg target genes *nkd* and *wf*, and a downregulation of the negative Wg target *arr* (Figure 8E). Moreover, in *als*-depleted cells, *ter94* reduction led to an amelioration of the changes in Wg target expression (Figure 8E), and to a partial restoration of Arm levels (Figure 8D, lane 12). In contrast, the Hh target gene *ptc* was not altered (Figure 8E). Overexpression of *ter94^RNAi^* in wing imaginal discs, however, caused pupal lethality. Because of this, it was difficult to analyze the adult wing phenotype caused by the combined depletion of *ter94* and *als*; because of *ter94*'s pleiotropic role, the analysis of the consequences of *ter94* depletion is challenging [38].

In addition, we found that overexpression of Ter94^HA^ suppressed the strong dominant-negative-like effect of Als^HA^ overexpression in the wing and the eye primordium (Figure 8F–8I), while overexpression of Ter94^HA^ could not suppress phenotypes caused by *als^RNAi^* (Figure S2M). This is consistent with the finding that overexpression of Ter94^HA^ alone did not cause any phenotype (Figure S2N); however, it suggests that an excess of Ter94^HA^ can neutralize the excess of the dominant-negative Als^HA^.

Together, these results suggest that Als functions downstream of GSK3β and downstream or at the level of E1 Ub ligase and that Als is required to antagonize Ter94 to allow normal Wg signaling. This is in agreement with our genetic epistasis experiments (Figure 5) and is corroborated by the further finding that overexpression of CSN6, an essential de-ubiquitinating enzyme and negative regulator of SCF/Slimb/βTRCP E3 Ub ligase [39], reverted the *als^RNAi^* phenotypes in the wing and the eye (Figure S11H–S11N). Of note, CSN6 overexpression suppressed the Wg signaling defects caused by Axin overexpression (Figure S11A and S11B), but did not suppress Wg signaling defects caused by Lgs^17E^ overexpression (Figure S11D and S11E).

### Human UBXN6 Is the Functional Ortholog of Als

**Figure 9:**
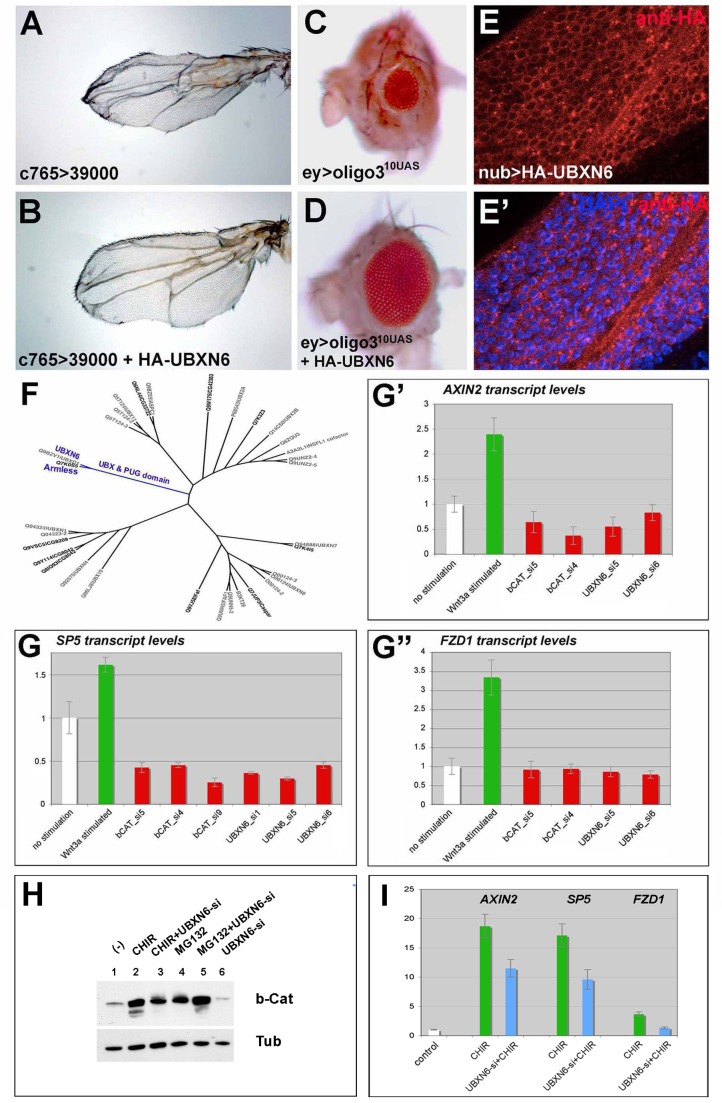
Human UBXN6 is the functional ortholog of Als. (A–D) Human UBXN6 expression could largely or completely rescue *als^RNAi^* wing and eye phenotypes. (E and E′) When expressed in wing imaginal discs ^HA^UBXN6 localization resembles that of ^HA^Als (cf. Figure S8A–S8E). (F) UBXN6 and Armless belong to the same protein subfamily of UBX domain proteins (blue). (G–G″) Expression of the WNT target genes *SP5*, *AXIN2*, and *FZD1* was upregulated upon stimulation of the pathway with mWnt3a (green bar), and was reduced upon siRNA against human *UBXN6* in HEK-293 cells. Depletion of β-Catenin served as a control. (H) β-Catenin levels were reduced upon siRNA against human *UBXN6* in HEK-293 cells (lane 6, cf. lane 1; and lane 3, cf. 2), whereas depletion of *UBXN6* had no effect on β-Catenin levels upon inhibition of the proteasome (MG132; lane 5, cf. lane 4). (I) WNT target genes were upregulated upon GSK3β inhibition (CHIR; green bars), and were reduced upon depletion of *UBXN6* (blue bars).

To address the evolutionary conservation of Als's function, we tested whether the human ortholog of Als, UBXN6, can substitute for Als in wing and eye development. Overexpression of ^HA^UBXN6 in imaginal discs could largely rescue the wing phenotypes and fully rescue the eye phenotypes caused by *als^RNAi^* (Figure 9A–9D). Further, we found that ^HA^UBXN6 localizes in cells of imaginal discs in a manner similar to ^HA^Als (Figure 9E and 9E′, cf. Figure S8A–S8E). The UBXN6 and Als proteins share 33% identity and belong to the same subfamily of UBX domain proteins, which is specified by the presence of an UBX and a PUG domain (Figure 9F). We next asked whether UBXN6 is required for Wnt signaling in human cells. We stimulated the Wnt pathway in HEK-293 cells with mouse Wnt3a (mWnt3a) while targeting *UBXN6* by small interfering RNA (siRNA). siRNA against *CTNNB1/*β-*Catenin* served as a control. To monitor the output of the Wnt pathway, we analyzed three established Wnt-responsive genes, *SP5*, *AXIN2*, and *FZD1*, by real-time PCR. All three targets were upregulated upon pathway stimulation (Figure 9G–9G″, green bars), and this response was abolished upon siRNA treatment of *UBXN6* (Figure 9G–9G″, red bars). The depletion of β-*Catenin* caused a similar downregulation of Wnt target gene expression. Further, we found β-Catenin levels reduced upon *UBXN6* depletion in HEK-293 cells (Figure 9H, lane 6, cf. lane 1). Similar to mWnt3a, the stimulation of the pathway by GSK3β inhibition caused an increase of Wnt target genes. Depletion of *UBXN6* reduced this effect (Figure 9I). These findings suggest that UBXN6 is a functional ortholog of Als and that UBXN6 might play a similar role for Wnt signaling in human cells.

## Discussion

A prevalent mechanism for controlling information flow in signaling pathways is the alteration of the protein levels of key components. In the Wg/Wnt pathway, the Arm/β-Catenin destruction complex targets Arm/β-Catenin for ubiquitination by the SCF/Slimb/βTRCP E3 Ub ligase, resulting in proteasomal degradation and low cytoplasmic levels of Arm/β-Catenin in the Wnt pathway off state. If the pathway is turned on, Slimb-mediated ubiquitination is prevented, thus rescuing Arm from its proteasomal fate and causing a concomitant increase in Arm protein levels [5]. Here we describe Als as a new component of this control system; we found that Als is required to prevent the degradation of Arm/β-Catenin.

### Als/UBXN6 Is a Novel Regulator of the Wnt/Wg Pathway

We identified *als* in a genome-wide in vivo RNAi screen in *Drosophila*. Because we did not succeed in isolating an EMS- or P-element-induced null allele and because another gene overlaps with *als*, we demanded, and obtained, particularly thorough evidence validating *als* gene function. (1) The *als^RNAi^* phenotypes could be reproduced by nine different *UAS-RNAi* transgenes encoding independent RNA target sites. Together with an extended off target analysis, we could rule out unintentional RNAi as a cause for the *als* phenotypes. (2) RNAi-mediated inhibition of *als* expression was ascertained by monitoring *als* mRNA expression via real-time PCR and antisense mRNA in situ hybridization. (3) Expression of ^HA^Als with different RNAi-insensitive rescue transgenes, as well as with its human ortholog UBXN6, rescued *als^RNAi^* phenotypes.

Our analyses show that *als* encodes an essential positive Wg signaling component. This conclusion is based on the following evidence. *als* depletion caused wings with notched wing margins and loss of sensory bristles, which is characteristic of impaired Wg signaling. The distal wing region is most sensitive to *als* levels, as is the case for other positive components of Wg signaling. In agreement with this, we found increased *als* expression in the central wing pouch, at least in earlier L3 larval stages. Stimulation of the Wg pathway in wing imaginal discs or Kc-167 cells caused higher *als* expression, suggesting that *als* can be positively controlled by Wg signaling. However, Als levels must be precisely controlled since already mild overexpression of *UAS-als* elicits a dominant-negative effect on Wg signaling. The function of *als* for Wg signaling is not restricted to the wing: also in other tissues, such as the thorax, eyes, legs, and the embryo, *als^RNAi^* phenotypes are identical to those seen when Wg signaling is disturbed. Also in human HEK-293 cells we found UBXN6/UBXD1, the ortholog of Als, to be required for Wnt signaling, and human ^HA^UBXN6 largely rescued the *als^RNAi^* phenotypes in *Drosophila*, which suggests their functional conservation. Depletion of *als* also enhanced Wg-sensitized phenotypes, further supporting the notion that its product is a Wg pathway component. Moreover, the expression of positively regulated Wg target genes is reduced or abolished upon loss of *als* function, while Wg-repressed target gene expression is ectopically activated. Importantly, while interfering with *als* function suppressed Wg signaling, it did not affect other pathways, such as Notch and Hh, Jak/Stat, or EGFR signaling. However, we cannot rule out that *als* is not required in another pathway in a different biological context. In humans, UBXN6 is reported to play a role in diverse scenarios: for example, it was shown to play a role in Caveolin turnover in human osteosarcoma U2OS cells [40]. This might indicate a broader role of UBXN6 in mammalians.

### Als: A Protector of Armadillo

Our data show that Als regulates Armadillo protein levels. Based on our epistasis experiments, Als acts downstream of Shaggy/GSK3β and upstream of the SCF/Slimb/βTRCP E3 Ub ligase, which is known to ubiquitinate Arm, a prerequisite for proteasomal degradation. Consistent with this, the degradation-resistant form of Arm, Arm^S10^, could completely bypass the requirement for *als*, in contrast to the wild-type form of Arm. This suggests that proteasomal degradation acts downstream of *als*; however, we would like to point out that this cannot be taken as an unambiguous proof. Importantly, depletion of *ubiquitin* and overexpression of CSN6, a negative regulator of SCF/Slimb/βTRCP E3 Ub ligase, could ameliorate the *als* phenotype (as well as phenotypes based on the overexpression of Axin or Shaggy, which overactivate the destruction complex, thus resulting in enhanced Arm degradation). In contrast, altering these factors did not ameliorate the Lgs^17E^ phenotype, which is caused by interfering more downstream in the Wg pathway. These findings suggest that *als* works upstream of proteasomal degradation. A further informative experiment was monitoring Wg pathway components with respect to protein levels: Arr, Fz, Axin, APC, Sgg, and Arm. The only change in the absence of Als function was Arm: its levels were strongly reduced upon *als* depletion. The effects on Arm levels could be due either to a direct effect on Arm or to an indirect effect on a negative component. Importantly, the rate-limiting factor Axin as well as other key negative components of the Arm/β-Catenin destruction complex were unaltered upon *als* depletion.

### Als Interacts with Ter94 ATPase

Some further mechanistic insight was obtained with our finding that Ter94 interacts in vitro and in vivo with Als. Interestingly, we found that Als-Ter94 localizes at the cell cortex, as was similarly observed for the Arm/β-Catenin destruction complex [41]. Our studies are consistent with earlier work that showed that the human ortholog of Ter94, p97, interacts with UBXN6 [42]. Ter94/p97/Cdc48 is a conserved and highly abundant AAA ATPase that was found to associate with SCF/Slimb/βTRCP E3 Ub ligases or proteasomal shuttle factors to mediate UPS-mediated protein degradation ([33],[42]–[45]; reviewed in [6],[34]). Specifications of the diverse activities of Ter94/p97 and the fate of its substrates are mainly exerted by UBX domain protein co-factors, which eventually either promote or hinder p97's function in protein turnover; an example of the latter involves the dissociation of the SCF/Slimb/βTRCP E3 Ub ligase complex, eventually leading to its inactivation [45]. Interestingly, it was recently reported that inactivation of the E3 ligase complex upon Wnt signaling is achieved by its dissociation from the destruction complex [5]. Based on our experiments and what is known about Ter94/p97, we suggest as a possible mechanism that Als antagonizes Ter94's positive effect on E3 ligase function, thereby rescuing Arm levels. We, however, did not observe increased protein levels of Slimb, Axin, Shaggy, or APC in our analyses; thus, our results favor a model in which Als antagonizes Ter94 to hinder the transfer of Arm to the proteasome by interfering with the SCF/Slimb/βTRCP E3 Ub ligase function or its assembly. Importantly, we did not find an interaction between Arm and Als. This is consistent with the finding that UBX domain family members lacking an UBA domain, such as UBXN6/Als, do not directly interact with substrate proteins [44], but are necessary for the activity or fate of the Ter94/p97.

Interestingly, Zhang et al. [38] found that *ter94* depletion affected the partial proteolysis of Ci. However, they observed neither any typical consequence of disturbed Hh signaling per se (i.e., no alteration of Hh target gene expression in genes such as *ptc*) nor any phenotypical consequence upon overexpression of a dominant negative form of Ter94 (i.e., aberrant wing patterning and growth typical for Hh signaling). This is consistent with our data that neither Ci target expression nor Hh signaling was affected upon *als* or *ter94* depletion (Figures S6G–S6J and 8E).

p97/Ter94 is known as a highly pleiotropic AAA ATPase associated with many cellular functions. Further, p97/Ter94 acts in multifaceted and large protein–protein complexes, and it is its regulatory co-factors, including UBX domain proteins, that render p97/Ter94 specific for a certain task in a particular cellular context. For example, p47/Shp1 is a co-factor of p97/Ter94 that blocks other co-factors from Ter94 binding [46],[47]. Interestingly, in our Kc-167 cell mass spectroscopy experiments, we found p47 in Ter94/Als protein complexes, but only in the absence of Wg stimulation (Text S1). On the other hand, we found *als* transcript and Als protein levels elevated upon Wg signaling (Figures 8A and S5D). These findings suggest a dynamic regulation of the Ter94 complex upon signaling inputs. The identification and functional analysis of all key components of the Als-Ter94 complex will be needed to obtain a refined insight into Als-Ter94's molecular mechanism.

### Concluding Remarks

Critically, our work spotlights an underappreciated facet in the control of the output of the entire canonical Wg/Wnt pathway—how Arm/β-Catenin is handed over to the proteasome— and the potential for regulating this step; our works also indicates that this step, in contrast to the conventional wisdom, is tunable. Our identification and characterization of the UBX protein Als as a positive regulator of Wg/Wnt signaling contributes to this layer of pathway control.

## Materials and Methods

### Drosophila Strains

#### *UAS*-*als^RNAi^* lines

*als* was identified in a genome-wide RNAi screen based on the Vienna Drosophila Resource Center (VDRC) RNAi library [48] with the VDRC line 28998 (II/CyO).

To further validate this phenotype we used the *UAS-RNAi* lines GD-39000 (Chromosome 3) and KK-105104 (Chromosome 2), both obtained from the VDRC. Seven further lines were generated by us, representing six different target regions that target either the coding sequence or the UTRs of *als* (Figure S1A). The algorithm used to design the RNAi target site of the 21-mer oligos [49] is indicated as superscript. All lines are based on Chromosome 3 site-specific insertion using the *attP-86Fb* line (FlyORF, [50]): *pNE3*::oligo2^Dharmacon (ORF)^ (target site: 5′-CCGCCAAAAGTATGAGGCCTA-3′), *pNE3*::oligo3^Dharmacon (ORF)^ (target site: 5′-CGGACGAAAGCCTGGATTTTA-3′), *pWAL20*::oligo3^Dharmacon (ORF)^ (target site: 5′-CGGACGAAAGCCTGGATTTTA-3′), *pWAL20*::oligo1^DSIR (ORF)^ (target site: 5′-GCAAGATACGGATGTCCAACA-3′), *pWAL20*::oligo2^Dharmacon (3′utr)^ (target site: 5′-CAGCGGTATTTGTCCCTTATA-3′), *pWAL20*::oligo2^Dharmacon (5′utr)^ (target site: 5′-CAAGGAAATCGAGGAGCTTAA-3′), *pWAL20*::oligo3^Dharmacon (5′utr)^ (target site: 5′-CGGCTATTCGTGGTCCATTTA-3′).

*pNE3*-based RNAi lines contain five copies of *UAS*; *pWAL20*-based RNAi lines contain ten copies of *UAS*. The *pNE3* vector was obtained from B. Haley and is a derivative of the *pUAST-attB* vector [49],[50]; the *pWAL20 (pWALIUM20)* vector was obtained from J. Ni (Transgenic RNAi Resource Project [TRiP], Drosophila RNAi Screening Center).

#### RNAi off target analysis

We analyzed the mRNA levels of computer-predicted, putative off targets of the VDRC lines upon expression of *UAS-VDRC-RNAi* transgenes in wing imaginal discs by real-time PCR. We did not find specific alterations of the putative off targets. For the generation of our *UAS-oligo-RNAi* transgenes, we employed the DRSC *off-target-finder* algorithm (16-mer allowed) to exclude off-target-prone RNAi target sites.

#### Genetic interaction

We used the *als* Exelixis deficiency line BL7894, Df(2R)Exel7157/CyO (homozygous lethal), to test the genetic ineraction with *als^RNAi^*.

#### Lines for overexpression: *UAS-als* lines

Transgenic lines are based on the ΦC31 transgenesis system, using the line ZH-86Fb attB as the landing site on Chromosome 3.

cDNA for the 1.327-kb long coding region of *als* was obtained by PCR on cDNA from L3 wing imaginal disc after total RNA extraction. A *Drosophila* Kozak consensus region was attached in front of the ATG. N- and C-terminally epitope-tagged versions are as follows: *UAS-als^HA^* (C-terminal triple HA-tag), *UAS-^HA^als* (N-terminal triple HA-tag), *UAS-^FLAG^als* (N-terminal double FLAG-tag), *UAS-^HA^als^oligo3-mut^* (N-terminal triple HA-tag); within the *oligo3* target site we introduced seven conservative basepair mutations by alternative codon usage (5′-CTGATGAGAGTCTAGACTTCA-3′).

#### Als protein domain deletion variants

Deletions of the PUG, UBX, and N-terminal domains were generated by overlap extension or conventional PCR.

#### *UAS-^HA^UBXN6* line

The coding region of human *UBXN6* was isolated by PCR on cDNA derived from HEK-293 cells after total RNA extraction.

#### Further transgenic lines for wing and eye analysis

The following lines were used: *UAS-Lgs^17E^* (K. Basler laboratory [K. B.]), *UAS-HLHmbeta* (FlyORF), *UAS-e(spl)m8* (FlyORF), *UAS-ras85D^RNAi^* (VDRC), *sal>smoI* (K. B.), *UAS*-*pan^RNAi^* (VDRC), *UAS-wingful/notum^RNAi^* (VDRC 45905), *UAS-shaggy/gsk3*β*^RNAi^* (VDRC 101538, TRiP BL 38293, 35364), *UAS-slimb^RNAi^* (TRiP BL 33898), *UAS*-*apc2^RNAi^* (NIG-FLY 3060R-3), *UAS-apc^RNAi^* (TRiP BL 51468), *UAS-axin^RNAi^* (TRiP BL 31705), *UAS-dsh* (FlyORF), *UAS-axin*, *UAS-shaggy*, *UAS-axin-GFP* [36], *UAS-shaggy^HA^* (FlyORF), *UAS-apc2^HA^* (FlyORF), *UAS*-*fz2^HA^* (FlyORF), *UAS-arrow^HA^* [17], *UAS-CG42306* (K. B.), *UAS-GFP* (K. B.), *UAS*-*arm* (K. B.); *UAS*-*arm^S10-Myc^* [31], sev-*wg* eye tester: yw, *ey-flp* (X chromosome; K. B.), *ey*-*Gal4*,*GMR-Gal4*/CyO (Chromosome 2; K. B.), *sev>y+>wg*/TM6b (Chromosome 3; K. B.), *UAS-diap1* (K. B.), *UAS-string*, *GMR>eiger*/CyO (K. B.), *UAS-Ter94^HA^* (FlyORF), *UAS-CSN6* (FlyORF), *UAS*-*ubip5E^RNAi^* (TRiP BL 38967), *UAS*-*ter94^RNAi^* (VDRC 24354, TRiP BL 32869).

#### Gal4 driver lines

The following lines were used: *nubbin (nub)-Gal4*, *c765*-*Gal4*, *MS1096*-*Gal4*, *apterous*-*Gal4*, *hedgehog (hh)*-*Gal4* (combined with *UAS-CD8-GFP*), *engrailed (en)-Gal4* (combined with *UAS-GFP*), *spalt (salE)*-*Gal4*, *vestigial (vg)181*-*Gal4*, *distalless (dll)*-*Gal4*, *pannier (pnr)-Gal4*, *eyeless (ey)*-*Gal4*, *GMR*-*Gal4*, *da*-*Gal4*).

#### Cell clone analysis

For cell clone analysis in wing imaginal discs (Figure 4) and adult wings, somatic clones were generated in animals of the following genotype: *yw, hsflp/+; +/+; actin5c>CD2>Gal4, UAS-GFP, UAS-forked^RNAi^/UAS-als^RNAi^* (VDRC line 39000 or line *oligo3^10UAS^*) or *UAS*-*als^HA^*. Heat shock flippase *(hsflp)* was induced 2 and 3.5 d after egg deposition (30 min, 37°C). Third instar larvae were fixed and stained for Wg target expression by standard immunohistochemical techniques. *UAS-forked^RNAi^* was used to mark adult cell clones (trichomes of the wing epithelium are shorter and adopt a *forked*-like shape).

#### Transcriptional reporter lines

The following lines were used: *wingless-lacZ, arrow-lacZ l(2)k08131*, *frizzled3-lacZ, distalless-lacZ, wingful-lacZ, e(spl)m8HLH.1.25-lacZ, NRE-eGFP* (TRiP BL 30728).

#### *als*-*lacZ* transgenic lines

4,978 kbp (HindIII-XbaI) and 2,927 kbp (StuI-XbaI) of *als*'s upstream genomic DNA (down to the start codon) were cloned into a *pattB-lacZ* vector. Wing imaginal disc expression of the lines was analyzed by X-Gal staining (brightfield microscopy) and by anti-βGal antibody staining, visualized by Alexa Fluor 594 secondary antibody (UV confocal microscopy).

### In Vivo Interaction of Als with Ter94 (BiFC Analysis)

Ter94-VNm9 (Ter94 fused to the N-terminal part of Venus-YFP) and Als-VC155 (Als fused to the C-terminal part of Venus-YFP) were co-expressed and analyzed for interaction by BiFC [34]. Direct interaction of two proteins causes a reconstitution of the two non-fluorescent YFP subfragments into a functional YFP, resulting in a yellow fluorescent signal.

### Immunohistochemistry of Imaginal Discs

#### Primary antibodies

mouse anti-β-Galactosidase (1∶2,000, Promega), rabbit anti-Arrow (1∶15,000, generous gift from S. DiNardo), guinea pig anti-Senseless (1∶800, gift from H. Bellen), rat anti-Distalless (1∶500, gift from S. Cohen), mouse anti-Wingless (1∶1,000, 4D4, Developmental Studies Hybridoma Bank [DSHB]), rabbit anti-E-Cadherin (1∶100, DSHB), mouse anti-Frizzled2 (1∶20, DSHB), mouse anti-Armadillo (1∶100, N2 7A1, DSHB); mouse anti-HA (1∶500, 6E2, Covance), rabbit anti-HA (1∶200, SC805, Santa Cruz Biotechnology), mouse anti-c-Myc (1∶200, 9E10, DSHB).

#### Secondary antibodies

Alexa Fluor 594 and 488 (1∶400, Molecular Probes) were used as secondary antibodies. Larval head pieces were fixed in 4% formaldehyde for 30 min at room temperature. Imaginal discs were mounted in Vectashield (Vector Laboratories).

### Imaging

Images of immunostainings were generated with a Zeiss Lsm710 confocal microscope using 40× and 60× oil objectives. Images were analyzed with ImageJ software. Adult fly wings were mounted in Euparal, and images were generated with a Zeiss Axioplan microscope.

### In Situ mRNA Hybridization

RNA probes were generated by run-off transcription of antisense mRNA with T7 RNA polymerase (Promega) from linearized plasmid templates with the PCR-amplified *als* coding region (full-length template as well as an EcoRV-truncated template, generating the first half of the coding sequence). Antisense mRNA was generated with a 3′ primer containing the T7 RNA polymerase binding sequence (5′-GAATTTAATACGACTCACTATAGG-3′).

### Real-time PCR

*UAS*-*als^RNAi^* was expressed under the control of *c765*-*Gal4* (Figure S1L) in wing imaginal discs, which were collected at the late third instar larval stage for total RNA isolation (RNeasy Kit, Qiagen, or TRI-Reagent, Sigma-Aldrich). 1 µg of total RNA was used as a template for reverse cDNA transcription (Superscript-RT-II, Roche). Quantitative PCR reactions were carried out with ¼ of the real-time PCR reaction and *als*-specific primers, 25 cycles, in the presence of SYBR Green (Roche), which enabled quantitative detection of the amplicons. Primers were designed by Roche Universal ProbeLibrary and are intron-spanning (*als* primer pair *a*: forward: ggaaaagacactatatgactgcaaact; reverse: aaggagcgggtgtatcattg; *als* primer pair *b*: forward: gaaaccatgtccaagattaagaagt; reverse: atgccgctgccagttaaa). Real-time PCR was carried out on an Eppendorf Realplex Mastercycler. mRNA levels were calculated with the comparative CT (threshold concentration) method and were normalized for three external standards (*actin5C*, *TBP*, and *gpdh*). For each RNAi condition, data were obtained from three biological replicates (i.e., three independent but identical *Gal4*×*UAS-RNAi* fly crosses), and real-time PCR was carried out in technical triplicates per experiment.

### *Drosophila* Cell Culture and Immunoprecipitation of Epitope-Tagged Proteins

Kc-167 cells were cultured in Schneider's Drosophila medium (Invitrogen) supplemented with 10% fetal calf serum and 1% penicillin/streptomycin at 25°C. For each experimental condition, 2× T75 (8 ml) culture flasks of 90% confluent Kc-167 cells were seeded at 60% confluency. 1 d after seeding, cells were transfected with the *UAS* transgenes (*UAS-als^HA^*, *UAS-ter94^HA^*, *UAS- ^FLAG^als, UAS- ^FLAG^*Δ*N-als*). *UAS-GFP* served as a control for transfection efficiency; empty *pUAST-attB* vector and *UAS-^FLAG^Gal4* vector served as negative controls; UAS transgenes were expressed under the control of *tubulin*-*Gal4*. DNA input per T75 flask was 0.5 µg of *tubulin-Gal4*, 0.75 µg of *UAS-GFP*, 0.5 µg of control vector, and 1.5 µg of the respective *UAS* transgene. Effectene (Qiagen) was used for transfection, according to the manufacturer's protocol. For stimulation of the Wg pathway, the supernatant of cells containing secreted Wg was added to the cell culture 24 h after transfection.

#### Immunoprecipitation

Cells were harvested 48 h post-transfection. Per each experimental condition, cells of two T75 flasks were pooled and lysed in lysis buffer (8× of pellet volume) containing 20 mM Na_2_H_2_PO_4_ (pH 7.4), 200 mM NaCl, 0.5% Nonidet P-40, 2 mM EDTA, 1 mM DTT, 1.5 mM Na_3_VO_4_, and protease inhibitor (Complete Mini; Roche). Cell suspension was dounced 20×, incubated 10 min at room temperature, and centrifuged with 13,000 rpm at 4°C. The supernatant (total cell lysates) was incubated either with rabbit anti-HA antibody (Abcam) or mouse anti-FLAG antibody (Sigma), conjugated to Protein-A Sepharose beads (Sigma), and allowed to rotate 3 h at 4°C. The beads were then collected (3,600 rpm, 10 min at 4°C), washed 3× with the lysis buffer, and transferred to a column to allow gravity flow. Protein complexes were eluted with 3×50 µl of glycine (0.2 M, pH 2.5) and neutralized with 50 µl of 8 M urea (containing 100 mM NH_4_CO_3_). The pH of the eluted protein complex was adjusted with NH_4_HCO_3_ to a final pH of 8.8 and stored at −20°C prior to sample preparation for mass spectroscopy (see below).

For Western blot analysis, protein complexes were denaturated and resolved on 4%–12% NuPAGE Bis-Tris Mini Gels (Invitrogen) and transferred to a nitrocellulose membrane. After blocking, the membrane was incubated with either anti-HA antibody (mouse, 1∶1,000, HA.11, Covance) or anti-FLAG M2 antibody (mouse, 1∶1,000, Sigma), followed by the respective secondary goat anti-mouse antibodies conjugated with horseradish peroxidase (1∶10,000). Signals were detected with ECL reagents (Amersham), followed by autoradiography. Membranes were stripped (Re-Blot Plus Strong Solution, Millipore), blocked, and reused for incubation with further primary antibodies (mouse anti-αTubulin, 1∶2,000, Sigma; rabbit anti-UBXD1, 1∶1,000; rabbit anti-Ter94 1∶1,000).

For dsRNA experiments, 45 µg of dsRNA (control *gfp*-dsRNA or *als*-dsRNA) was incubated with 7.5×10^6^ cells per condition for 1 h before transfer into T75 flasks. Cells were harvested 3.5 d after seeding. The GSK3β inhibitor CHIR 99021 was added to the cells 20 h before cell harvest (12 µM), the E1 ligase inhibitor PYR-41 (Calbiochem) was added 2 h before cell harvest (50 µM), and the proteasome inhibitor MG-132 was added 6 h before cell harvest (25 µM). Primary antibodies for Western blots after immunprecipitation were rabbit anti-Phospho-β-Catenin (Ser33/37) (1∶750, Cell Signalling), mouse anti-Armadillo (1∶50, N27A1, DSHB).

### Mass Spectrometry Analysis of Epitope-Tagged Als and Ter94

#### Sample preparation for mass spectrometry

Proteins isolated via immunoprecipitation against the hemagglutinin-tag were reduced with 5 mM TCEP and treated with 10 mM iodoacetamide to modify cysteine residues. Tryptic digestion was performed overnight using 2–5 µg of trypsin per sample. Digested peptides were purified by reverse phase C-18 chromatography (Sep-PaK, Waters). For mass spectrometry analysis, samples were resuspended in buffer containing 5% acetonitrile and 0.2% formic acid.

#### Mass spectrometry parameters and data analysis

For each sample, 4 µl was loaded on a LTQ-Orbitrap XL ETD mass spectrometer (Thermo Fisher Scientific). The instrument was coupled to an Eksigent NanoLC system. Samples were automatically injected into a 10-µl sample loop and loaded onto an analytical column that was packed in-house with Magic C18 AQ beads (3 µm, 200 Å, Bischoff Chromatography) and was 8 cm in length, with an internal diameter of 75 µm. Peptide mixtures were delivered to the analytical column at a flow rate of 500 nl/min (3% acetonitrile, 0.2% formic acid) for 16 min and then eluted using a gradient of acetonitrile (3%–35%; 0.53%/min) with 0.2% formic acid at a flow rate of 250 nl/min. The samples were measured in a survey scan from 300 to 2,000 amu, followed by six data-dependent LC-MS/MS scans with dynamic exclusion (isolation width 2 amu, three repeat counts, exclusion list size 500, dynamic exclusion duration 60 s). LC-MS/MS spectra were assigned to peptide sequences using Mascot software, version 2.4 (Matrix Science). All peptide assignments where the Mascot ion score was greater than the homology score and where the Mascot expect score was smaller than 0.05 were accepted.

### Human Cell Culture

HEK-293 cells were seeded into a 96-well plate (40,000 cells/well). 1 d after seeding cells were transfected (Lipofectamine 2000, Invitrogen) with siRNAs targeting human β-*Catenin*, human *UBXN6*, or a negative control siRNA (Qiagen FlexiTube siRNA). The siRNA concentration per well was 0.05 µM. Co-transfected *histone2B*-*RFP* served as the control of the transfection efficiency. The Wnt pathway was stimulated by mWnt3a from co-transfected *pcDNA3::mWnt3a*. Each experimental condition was made in triplicate. 48 h after transfection, cells were harvested, and an aliquot was used for real-time PCR (1) to analyze the siRNA-mediated knockdown of β-*Catenin* or *UBXN6* and (2) to analyze the transcriptional level of the Wnt targets *SP5*, *AXIN2*, and *FRIZZLED-1*. To analyze β-Catenin levels, siRNA (Qiagen FlexiTube siRNA, Thermo Scientific SMART pool) was applied two times (the second siRNA application was done 1.5 d after the first siRNA application), and cells were harvested 86 h after the first transfection.

## Supporting Information

Figure S1 **Validation of the** ***als^RNAi^*** **phenotype by different RNAi target sites.** (A) Location of 16 different siRNA target sites encoded by 16 RNAi lines with respect to the *als* cDNA region. The 16 different target sites are based on three different algorithms (VDRC, three different sites; Dharmacon, nine different sites; DSIR, four different sites). Nine of 16 target sites caused similar wing notching phenotypes (red bars in [A], similar phenotypes in [B–J]). Impairing Wg signaling via the overexpression of a dominant-negative pathway component, Lgs^17E^, causes a phenotype reminiscent of *als* depletion (K). Six target sites did not result in any phenotype (blue bars in [A]), and one target site caused a phenotype completely different from the nine other positive target sites, which we attribute to an off-target effect (OTE; purple bar in [A]). (L) Real-time PCR shows that ubiquitous expression of *UAS*-*als^RNAi^* by *c765-Gal4* effectively reduced *als* transcript levels in wing imaginal discs as monitored by two different real-time PCR primer pairs for *als* (*a* and *b* in the bar diagram). The degree of the mRNA knockdown correlates with the strength of the phenotype (cf. Figure 1B–1E). (M) Expression of *als^RNAi-39000^* with *c765-Gal4* does not cause a phenotype. (N) Haplo-deficiency for *als* causes wing notches in combination with *c765>als^RNAi-39000^* expression.
(TIF)

Figure S2 **Als can rescue** ***als^RNAi^*** **phenotypes.** (A) Overexpression of Als in wing imaginal discs often caused no phenotype, or mild notches at elevated expression (inset). (B) Overexpression of C-terminally epitope-tagged Als (Als^HA^) caused wings with strong notches. (C) Targeting the 5′ UTR of endogenous *als* by *als^oligo3_5′utr^* caused wings with notches. (D) Overexpression of N-terminally tagged Als (^HA^Als), which lacks UTRs, could rescue the *als^RNAi-utr^* phentoype. (E) Expression of *als^RNAi^*, which targets the ORF of endogenous *als* (*als^oligo3_10UAS_ORF^*), caused wings with marginal notches or a small eye phenotype (G). These phenotypes could largely or completely be rescued by the overexpression of an RNAi-insensitive version of ^HA^Als, which contains a mutated *oligo3_ORF* target site (F and H). Expression of *UAS*-*GFP* or *UAS-CG42306* did not rescue the *als^RNAi^* phenotype (I and J). Expression of *UAS-CG42306* did not cause a phenotype in the wing (K) and caused legs devoid of the entire tarsal region (L). (M) Ter94^HA^ overexpression did not rescue the *als^RNAi^* phenotype, and did not show any phenoype in the wing (N).
(TIF)

Figure S3 **Genetic interaction of** ***als*** **with the cell cycle and Wg signaling.** (A–F) Underproliferation, not apoptosis, underlies the *als^RNAi^* wing phenotype. Overexpression of a negative Wg pathway component, Lgs^17E^ (A), and of *als^RNAi^* (D) causes wing margin notches. Inhibition of apoptosis by overexpression of Diap1 could not rescue the wing phenotypes (B and E), whereas acceleration of the cell cycle by overexpression of String could rescue the wing phenotypes (C and F). (G–J″) *als* interacts with the Wg pathway. Reduction of Wg signaling (G–J) was provoked by (G) overexpression of a dominant-negative mutant form of Legless, Lgs^17E^, (H) depletion of *pangolin (pan)*, (I) overexpression of Shaggy (Sgg), or (J) overexpression of Axin. Simultaneously depleting *als* (G″–J″) leads to an enhancement of the notched wing margin phenotype observed with depleting *als* alone (G′–J′).
(TIF)

Figure S4 ***als*** **depletion in the thorax and legs.** (A–H) *als^RNAi^* expression in the notum, the primordium of the thorax, by *pannier-Gal4*, which is active in the mesothorax (E). *als* depletion causes mild thoracic clefts (B and C), a slight polarity defect of thoracic bristles, reduced or lost dorsocentral microchaete, and an overall shortening of the thorax and scutellum (B, C, F, and G). These phenotypes are reminiscent of impaired Wg signaling, for example, based on *pan^RNAi^* expression (D) or based on Lgs^17E^ overexpression (H). (I–K) *als^RNAi^* expression in the leg primordium by *distalless*-*Gal4* caused, similarly to impaired Wg signaling, a shortening of tarsal segments and a dorsolateral shift of the sex combs (J, K, L, and M), along with a significant dorsalization (K and M). (N) Impaired Notch signaling caused a loss of segmental junctions along the tarsus, resulting in a complete fusion of the tarsal segments. (O–Y) Analysis of impaired *als* function in the embryo. Expression of *als^RNAi^* or the dominant-negative-like Als^HA^ by *da*-*Gal4* ([S] *als^RNAi^ lines: oligo2_3′utr*; [T] *oligo3^10UAS^*) caused shorter cuticles than in control embryos, with ventral denticle belts partially fused (S–V, X, and Y). Expression of *arm^RNAi^* (P and Q) or *UAS-Lgs^17E^* by *da*-*Gal4* (R and W) caused similar phenotypes.
(TIF)

Figure S5 ***als*** **is expressed in the center of the wing pouch.** (A and B) *als* in situ hybridization reveals a pronounced expression of *als* in the center of the wing pouch and a low level of expression in the remaining part of the wing disc. (B) (inset) Expression of *UAS*-*als^RNAi^* by *hh*-*Gal4* in the P-compartment (arrows) of wing imaginal discs caused a strong decrease in *als* expression. (C) Reporter expression from a genomic *als*-*lacZ* transgene is similar to *als* expression visualized by in situ hybridization. (D) Activation of Wg signaling by the overexpression of either *UAS*-*wg* or *UAS*-*arm^S10^* with *nubbin*-*Gal4* at 29°C in wing imaginal discs was monitored by the upregulation of the Wg targets *wingful (wf)*, *naked (nkd)*, and *senseless (sens)* (bars 2–7). Similarly, activation of Wg signaling caused an upregulation of *als* expression (bars 8 and 9). Als was upregulated upon stimulation of the Wg pathway by inhibiting GSK3β in Kc-167 cells (detected by anti-UBXN6 antibody).
(TIF)

Figure S6 ***als*** **does not interfere with Notch or Hedghog signaling.** *m8-lacZ* expression at the D/V boundary of wing imaginal discs (A) is not altered upon *als* depletion (A′ and A″). NRE-GFP expression is not altered upon *als* depletion (inset of A and A″). *vg-BE-lacZ* expression (B) is not altered upon *als* depletion (B′ and B″). (C) Overexpression of E(spl), the nuclear mediator of Notch signaling, causes reduction and loss of wing veins. (D) E(spl) co-expressed with *als^RNAi^* resulted in wings with lost veins combined with wing notches. (E) Overexpression of HLHmbeta, a positive target of Notch signaling, abolishes wing veins. (F) HLHmbeta co-expressed with *als^RNAi^* resulted in veinless wings with marginal notches. (G) Overexpression of SmoI constitutively activates Hh signaling, which results in overgrown wings and ectopic veins. (H) Ectopic Hh signaling combined with *als^RNAi^* resulted in a wing size reduction predominantly of distal tissue, without altering the phenotypes symptomatic for elevated Hh signaling, like overgrowth along the A/P axis or ectopic veins. (I and J) *ptc-lacZ* expression is not altered upon depletion of *als*. (K) Ectopic expression of Upd, the ligand of the Jak/Stat pathway, caused overgrown eyes, which was not altered upon co-expression of *als^RNAi^* (L). (M) Impaired Ras/EGFR signaling caused slightly smaller wings lacking wing veins; co-expression of *als^RNAi^* resulted in veinless wings with marginal notches (N).
(TIF)

Figure S7 ***als*** **depletion does not affect Wg expression.** (A–F′) *wg*-*lacZ* and Wg protein expression is not altered upon expression of *als^RNAi^*. Adult phenotypes corresponding to the respective genetic manipulations during wing disc development are shown for line 39000 (A–C), line *oligo3^10UAS^* (D–E), and line *oligo3^10UAS^* (F–G). (F–G) Upon strong RNAi expression by *hh-Gal4* in the P-compartment (green, GFP expression), the P-compartment strongly undergrows, yet shows normal Wg expression.
(TIF)

Figure S8 **Als localizes adjacent to the apicolateral cell membrane.** ^HA^Als expressed with *nubbin*-*Gal4* in the wing pouch localizes in the cytoplasm adjacent to the cell membrane (A–E″). ^HA^Als localization overlaps to a large extent with that of E-Cadherin (C′ and C″), Arm (D′ and D″), and Fz2 (E′ and E″). Cell nuclei are blue (DAPI in the color-merged pictures [B′, C″, D″, and E″]).
(TIF)

Figure S9 **The PUG and the coiled-coil domains are essential for Als function.** Expression of *als^RNAi^* in the wing primordium with *nubbin-Gal4* (A) or with *181-Gal4* (B and C) causes nicked wing margins. Co-expression of Als^ΔPUG^ (D–F) or Als^ΔN^ (G–I) did not alter the *als^RNAi^* phenotype, whereas Als^ΔUBX^ rescued the *als^RNAi^* phenotype (J–L) comparably to full-length Als (insets of J–L). cc (coiled coil domain, within the N-terminal region of Als).
(TIF)

Figure S10 **Negative pathway components are not altered upon** ***als*** **depletion.** (A–D′) Axin-GFP expression levels are not changed upon depletion of *als* (line 39000 [C–D′, cf. A–B′]). Expression of Apc2^HA^ (E–H) and Shaggy^HA^ (I–L) is not altered upon *als* depletion (line 39000). (M–P) Expression levels of endogenous Fz2 (anti-Fz2-antibody staining) and Arrow^HA^ were not altered upon depletion of *als* (line *oligo3^10UAS^*, 29°C). Confocal pictures were taken at 40× magnification/2.5× zoom, except (A, A′, C, and C′): 40× magnification, 1× zoom; (E–L, O, and P): anti-HA antibody staining.
(TIF)

Figure S11 **Als acts upstream of Arm's proteasomal degradation.** The overexpression of CSN6 suppressed *als* ^RNAi^ phenotypes (H, K, and N, cf. G, J, and M) as well as a Wg loss-of-function phenotype caused by Axin overexpression (B, cf. A), but not as well as a Wg loss-of-function phenotype caused by the overexpression of Lgs^17E^ (E, cf. D). Similarly, depletion of *ubiquitin* could suppress phenotypes based on *als* ^RNAi^ (I, L, and O, cf. G, J, and M) and Axin overexpression (C, cf. A), but could not ameliorate the Lgs^17E^ overexpression phenotype (F, cf. D).
(TIF)

Text S1 **Supporting information.**
(DOCX)

## Abbreviations

A/P: anteroposterior
BiFC: bimolecular fluorescence complementation
D/V: dorsoventral
DSHB: Developmental Studies Hybridoma Bank
dsRNA: double-stranded RNA
LC-MS/MS: liquid chromatography–tandem mass spectrometry
mWnt3a: mouse Wnt3a
NRE: Notch responsive element
RNAi: RNA interference
siRNA: small interfering RNA
TRiP: Transgenic RNAi Resource Project
UPS: ubiquitin-proteasome system
VDRC: Vienna Drosophila Resource Center

## References

[1] Sharma RP, Chopra VL (1976) Effect of the Wingless (wg1) mutation on wing and haltere development in *Drosophila melanogaster* . Dev Biol 48: 461–465. 815114 10.1016/0012-1606(76)90108-1

[2] Rijsewijk F, Schuermann M, Wagenaar E, Parren P, Weigel D, Nusse R (1987) The *Drosophila* homolog of the mouse mammary oncogene *int-1* is identical to the segment polarity gene *wingless* . Cell 50: 649–657. 3111720 10.1016/0092-8674(87)90038-9

[3] Klaus A, Birchmeier W (2008) Wnt signalling and its impact on development and cancer. Nat Rev 8: 387–398. 10.1038/nrc2389 18432252

[4] Clevers H, Nusse R (2012) Wnt/β-Catenin signalling and disease. Cell 149: 1192–1205. 22682243 10.1016/j.cell.2012.05.012

[5] Li VSW, Ng SS, Boersema PJ, Low TY, Karthaus WR, et al (2012) Wnt signalling through inhibition of b-catenin degradation in an intact Axin1 complex. Cell 149: 1245–1256. 22682247 10.1016/j.cell.2012.05.002

[6] Schuberth C, Buchberger A (2008) UBX domain proteins: major regulators of the AAA ATPase Cdc48/p97. Cell Mol Life Sci 65: 2360–2371. 18438607 10.1007/s00018-008-8072-8 PMC11131665

[7] Neufeld TP, de la Cruz AFA, Johnston LA, Edgar BA (1998) Coordination of growth and cell division in the Drosophila wing. Cell 93: 1183–1193. 9657151 10.1016/s0092-8674(00)81462-2

[8] Couso JP, Bishop SA, Martinez Arias A (1994) The Wingless signalling pathway and the patterning of the wing margin in *Drosophila* . Development 120: 621–636. 8162860 10.1242/dev.120.3.621

[9] Giraldez AJ, Cohen SM (2003) Wingless and Notch signalling provide cell survival cues and control cell proliferation during wing development. Development 130: 6533–6543. 14660542 10.1242/dev.00904

[10] Phillips RG, Whittle JRS (1993) *Wingless* expression mediates determination of peripheral nervous system elements in late stages of *Drosophila* wing disc development. Development 118: 427–438. 8223270 10.1242/dev.118.2.427

[11] Neumann CJ, Cohen SM (1996) *Sternopleural* is a regulatory mutation of *wingless* with both dominant and recessive effects on larval development of *Drosophila melanogaster* . Genetics 142: 1147–1155. 8846894 10.1093/genetics/142.4.1147 PMC1207114

[12] Couso JP, Bate M, Martinez Arias A (1993) A *wingless* dependent polar coordinate system in *Drosophila* imaginal discs. Science 259: 484–489. 8424170 10.1126/science.8424170

[13] Brunner E, Peter O, Schweizer L, Basler K (1997) *pangolin* encodes a Lef-1 homologue that acts downstream of Armadillo to transduce the Wingless signal in *Drosophila* . Nature 385: 829–833. 9039917 10.1038/385829a0

[14] Blair SS (1996) Notch and Wingless signals collide. Science 271: 1822–1823. 8596948 10.1126/science.271.5257.1822

[15] Sato A, Kojima T, Ui-Tei K, Miyata Y, Saigo K (1999) *Dfrizzled-3*, a new Drosophila Wnt receptor, acting as an attenuator of Wingless signalling in *wingless* hypomorphic mutants. Development 126: 4421–4430. 10498678 10.1242/dev.126.20.4421

[16] Sivasankaran R, Calleja M, Morata G, Basler K (2000) The Wingless target gene Dfz3 encodes a new member of the Drosophila Frizzled family. Mech Dev 91: 427–431. 10704878 10.1016/s0925-4773(99)00313-5

[17] Wehrli M, Dougan ST, Caldwell K, O'Keefe L, Schwartz S, et al (2000) *arrow* encodes an LDL-receptor-related protein essential for Wingless signalling. Nature 407: 527–530. 11029006 10.1038/35035110

[18] Kramatschek B, Campos-Ortega JA (1994) Neuroectodermal transcription of the Drosophila neurogenic genes E(spl) and HLH-m5 is regulated by proneural genes. Development 120: 815–826. 7600959 10.1242/dev.120.4.815

[19] Furriols M, Bray S (2001) A model Notch response element detects suppressor of Hairless-dependent molecular switch. Curr Biol 11: 60–64. 11166182 10.1016/s0960-9822(00)00044-0

[20] Kim J, Sebring A, Esch JJ, Kraus ME, Vorkwerk K, et al (1996) Integration of positional signals and regulation of wing formation and identity by *Drosophila vestigial* gene. Nature 382: 133–138. 8700202 10.1038/382133a0

[21] Diaz-Benjumea FJ, Cohen SM (1995) Serrate signals through Notch to establish a Wingless-dependent organizer at the dorsal/ventral compartment boundary of the Drosophila wing. Development 121: 4215–4225. 8575321 10.1242/dev.121.12.4215

[22] Rulifson EJ, Blair SS (1995) Notch regulates wingless expression and is not required for reception of the paracrine wingless signal during wing margin neurogenesis in *Drosophila* . Development 121: 2813–2824. 7555709 10.1242/dev.121.9.2813

[23] de Celis JF, Garcia-Bellido A, Bray SJ (1996) Activation and function of Notch at the dorsal-ventral boundary of the wing imaginal disc. Development 122: 359–369. 8565848 10.1242/dev.122.1.359

[24] Brunner E, Brunner D, Weimin F, Hafen E, Basler K (1999) The dominant mutation *Glazed* is a gain-of-function allele of *wingless* that, similar to loss of APC, interferes with normal eye development. Dev Biol 206: 178–188. 9986731 10.1006/dbio.1998.9136

[25] Bänziger C, Soldini D, Schütt C, Zipperlen P, Hausmann G, et al (1996) Wntless, a conserved membrane protein dedicated to the secretion of Wnt proteins from signalling cells. Cell 125: 509–522. 16678095 10.1016/j.cell.2006.02.049

[26] Kramps T, Peter O, Brunner E, Nellen D, Froesch B, et al (2002) Wnt/Wingless signalling requires BCL9/Legless-mediated recruitment of Pygopus to the nuclear beta-Catenin-TCF complex. Cell 109: 47–60. 11955446 10.1016/s0092-8674(02)00679-7

[27] Geuking P, Narasimamurthy R, Basler K (2005) A genetic screen targeting the tumor necrosis factor/Eiger signalling pathway: identification of *Drosophila* TAB2 as a functionally conserved component. Genetics 171: 1683–1694. 16079232 10.1534/genetics.105.045534 PMC1456095

[28] Giraldez AJ, Copley RR, Cohen SM (2002) HSPG modification by the secreted enzyme Notum shapes the Wingless morphogen gradient. Dev Cell 2: 667–676. 12015973 10.1016/s1534-5807(02)00180-6

[29] Gerlitz O, Basler K (2002) Wingful, an extracellular feedback inhibitor of Wingless. Genes Dev 16: 1055–1059. 12000788 10.1101/gad.991802 PMC186249

[30] Jiang J, Struhl G (1998) Regulation of the Hedgehog and Wingless signaling pathways by the F-box/WD40-repeat protein Slimb. Nature 391: 493–496. 9461217 10.1038/35154

[31] Pai LM, Orsulic S, Bejsovec A, Peifer M (1997) Negative regulation of Armadillo, a Wingless effector in *Drosophila* . Development 124: 2255–2266. 9187151 10.1242/dev.124.11.2255

[32] Doerks T, Copley RR, Schultz J, Ponting CP, Bork P (2002) Systematic identification of novel protein domain families associated with nuclear functions. Genome Res 12: 47–56. 11779830 10.1101/gr.203201 PMC155265

[33] Pinter M, Jekely G, Szepesi RJ, Farkas A, Theopold U, et al (1998) Ter94, a *Drosophila* homolog of the membrane fusion protein CDC48/p97, is accumulated in nonproliferating cells: in the reproductive organs and in the brain of the imago. Insect Biochem Mol Biol 28: 91–98. 9639875 10.1016/s0965-1748(97)00095-7

[34] Meyer H, Bug M, Bremer S (2012) Emerging functions of the VCP/p97 AAA-ATPase in the ubiquitin system. Nat Cell Biol 14: 117–123. 22298039 10.1038/ncb2407

[35] Bischof J, Bjoerklund M, Furger E, Schertel C, Taipale J, et al (2013) A versatile platform for creating a comprehensive UAS-ORFeome library in *Drosophila* . Development 140: 2434–2442. 23637332 10.1242/dev.088757

[36] Fiedler M, Mendoza-Topas C, Rutherford T, Miesczczanek J, Bienz M (2011) Dishevelled interacts with the DIX domain polymerization interface of Axin to interfere with its function in down-regulating β-Catenin. Proc Natl Acad Sci U S A 108: 1937–1943. 21245303 10.1073/pnas.1017063108 PMC3033301

[37] Lange V, Picotti P, Domon B, Aebersold R (2008) Selected reaction monitoring for quantitative proteomics: a tutorial. Mol Syst Biol 4: 222. 18854821 10.1038/msb.2008.61 PMC2583086

[38] Zhang Z, Lv X, Yin WC, Zhang X, Feng J, et al (2013) Ter94 ATPase complex targets k11-linked ubiquitinated Ci to proteasomes for partial degradation. Dev Cell 25: 636–644. 23747190 10.1016/j.devcel.2013.05.006

[39] Ullah Z, Buckley MS, Arnosti DN, Henry RW (2007) Retinoblastoma protein regulation by the COP9 signalosome. Mol Biol Cell 18: 1179–1186. 17251548 10.1091/mbc.E06-09-0790 PMC1838975

[40] Ritz D, Vuk M, Kirchner P, Bug M, Schütz S, et al (2011) Endolysosomal sorting of uqiquitinated caveolin-1 is regulated by VCP/p97 and UBXD1 and impaired by VCP disease mutations. Nat Cell Biol 13: 1116–1123. 21822278 10.1038/ncb2301 PMC3246400

[41] Stamos JL, Weis WI (2013) The β-Catenin destruction complex. Cold Spring Harb Perspect Biol 5: a007898. 23169527 10.1101/cshperspect.a007898 PMC3579403

[42] Madsen L, Andersen KM, Prag S, Moos T, Semple CA, et al (2008) Ubxd1 is a novel co-factor of the human p97 ATPase. Int J Biochem Cell Biol 40: 2927–2942. 18656546 10.1016/j.biocel.2008.06.008

[43] Ghislain M, Dohmen RJ, Lévy F, Varshavsky A (1996) Cdc48p interacts with Ufd3p, a WD repeat protein required for ubiquitin-mediated proteolysis in *Saccharomyces cerevisiae* . EMBO J 15: 4884–4899. 8890162 PMC452226

[44] Beskow A, Grimberg KB, Bott LC, Salomons FA, Dantuma NP, et al (2009) A conserved unfoldase activity for the p97 AAA-ATPase in proteasomal degradation. J Mol Biol 394: 732–746. 19782090 10.1016/j.jmb.2009.09.050

[45] Yen JL, Flick K, Papagiannis CV, Mathur R, Tyrrell A, et al (2012) Signal-induced disassembly of the SCF ubiquitin ligase complex by CDC48/p97. Mol Cell 48: 288–297. 23000173 10.1016/j.molcel.2012.08.015 PMC3483439

[46] Kondo H, Rabouille C, Newman R, Levine TP, Pappin D, et al (1997) p47 is a cofactor for p97-mediated membrane fusion. Nature 388: 75–87. 9214505 10.1038/40411

[47] Bruderer RM, Brasseur C, Meyer HH (2004) The AAA ATPase p97/VCP interacts with its alternative co-factors, Ufd1-Npl4 and p47, through a common bipartite binding mechanism. J Biol Chem 279: 49609–49615. 15371428 10.1074/jbc.M408695200

[48] Dietzl G, Chen D, Schnorrer F, Su KC, Barinova Y, et al (2007) A genome-wide transgenic RNAi library for conditional gene inactivation in *Drosophila* . Nature 448: 151–156. 17625558 10.1038/nature05954

[49] Haley B, Hendrix D, Trang V, Levine M (2008) A simplified miRNA-based gene silencing method for *Drosophila melanogaster* . Dev Biol 321: 482–490. 18598689 10.1016/j.ydbio.2008.06.015 PMC2661819

[50] Bischof J, Maeda RK, Hediger M, Karch F, Basler K (2007) An optimized transgenesis system for *Drosophila* using germ-line-specific φC31 integrases. Proc Natl Acad Sci U S A 104: 3312–3317. 17360644 10.1073/pnas.0611511104 PMC1805588

