# Supplementary figures and images for: Protection of Armadillo/β-Catenin by Armless, a Novel Positive Regulator of Wingless Signaling
Source: PLoS Biol. 2014 Nov 4;12(11):e1001988. doi: 10.1371/journal.pbio.1001988 (PMC4219662; doi:10.1371/journal.pbio.1001988)

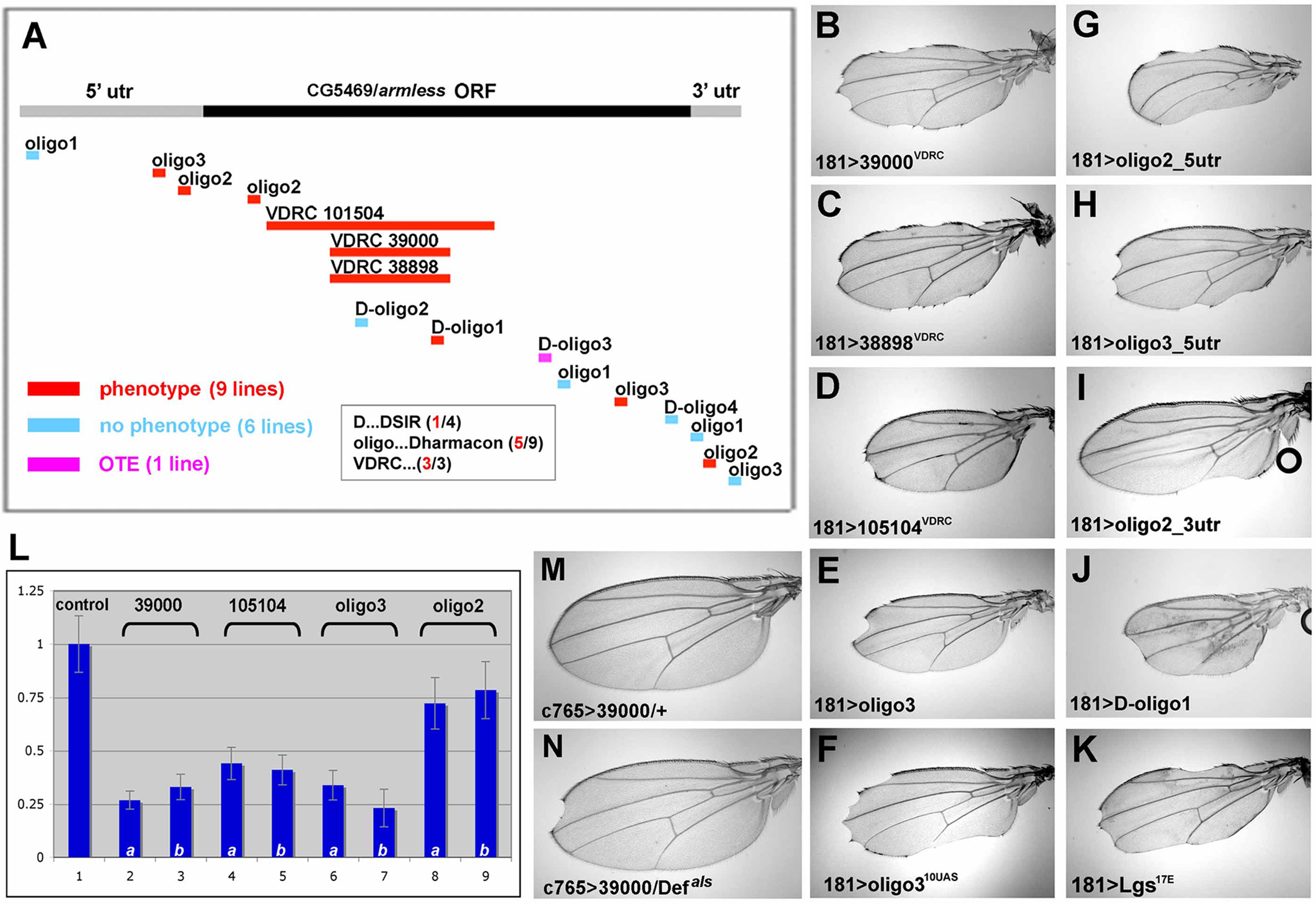

Supplement: Figure S1 — Validation of the alsRNAi phenotype by different RNAi target sites. (A) Location of 16 different siRNA target sites encoded by 16 RNAi lines with respect to the als cDNA region. The 16 different target sites are based on three different algorithms (VDRC, three different sites; Dharmacon, nine different sites; DSIR, four different sites). Nine of 16 target sites caused similar wing notching phenotypes (red bars in [A], similar phenotypes in [B–J]). Impairing Wg signaling via the overexpression of a dominant-negative pathway component, Lgs17E, causes a phenotype reminiscent of als depletion (K). Six target sites did not result in any phenotype (blue bars in [A]), and one target site caused a phenotype completely different from the nine other positive target sites, which we attribute to an off-target effect (OTE; purple bar in [A]). (L) Real-time PCR shows that ubiquitous expression of UAS-alsRNAi by c765-Gal4 effectively reduced als transcript levels in wing imaginal discs as monitored by two different real-time PCR primer pairs for als (a and b in the bar diagram). The degree of the mRNA knockdown correlates with the strength of the phenotype (cf. Figure 1B–1E). (M) Expression of alsRNAi-39000 with c765-Gal4 does not cause a phenotype. (N) Haplo-deficiency for als causes wing notches in combination with c765>alsRNAi-39000 expression. (TIF) [file pbio.1001988.s001.tif]

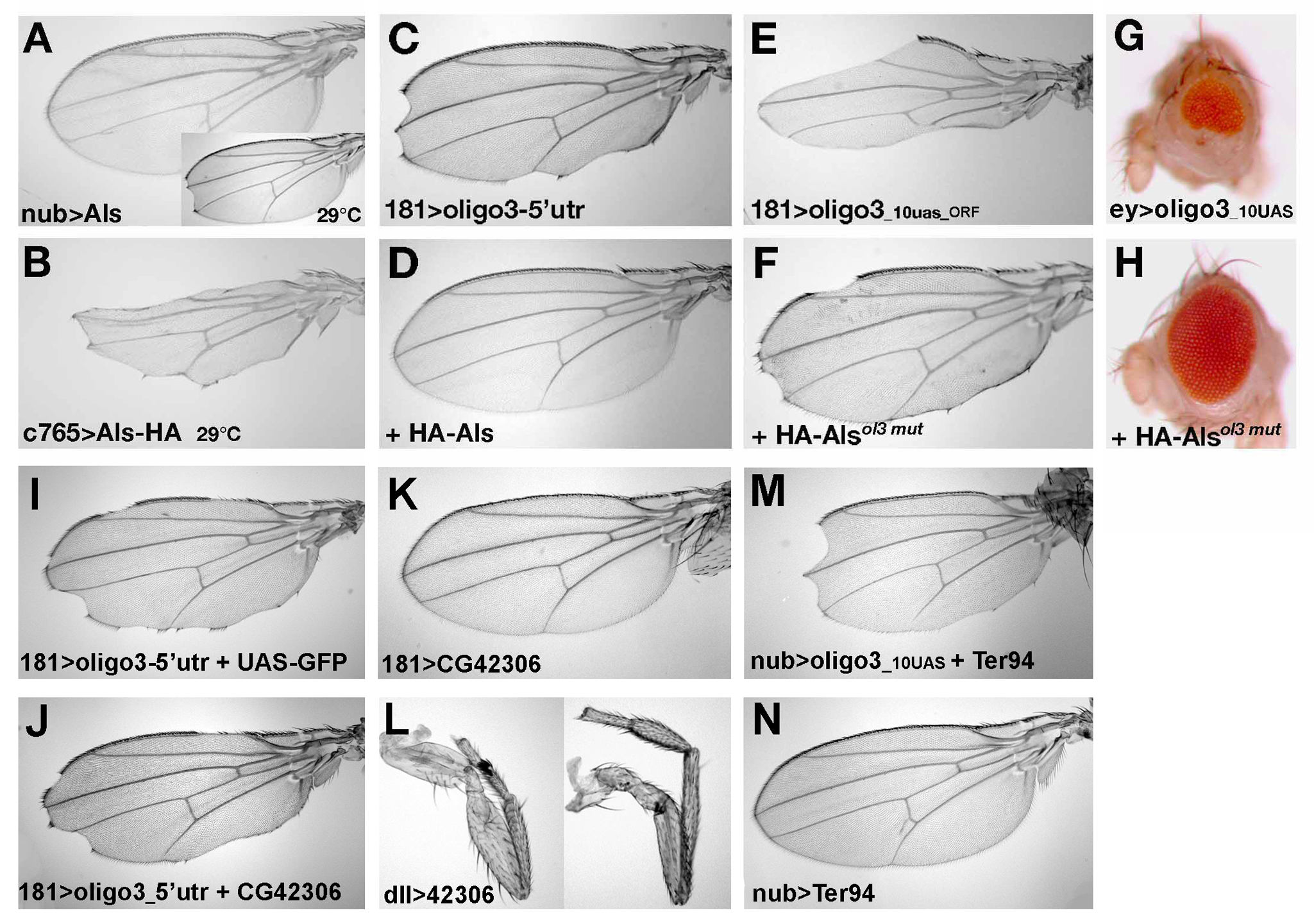

Supplement: Figure S2 — Als can rescue alsRNAi phenotypes. (A) Overexpression of Als in wing imaginal discs often caused no phenotype, or mild notches at elevated expression (inset). (B) Overexpression of C-terminally epitope-tagged Als (AlsHA) caused wings with strong notches. (C) Targeting the 5′ UTR of endogenous als by alsoligo3_5′utr caused wings with notches. (D) Overexpression of N-terminally tagged Als (HAAls), which lacks UTRs, could rescue the alsRNAi-utr phentoype. (E) Expression of alsRNAi, which targets the ORF of endogenous als (alsoligo3_10UAS_ORF), caused wings with marginal notches or a small eye phenotype (G). These phenotypes could largely or completely be rescued by the overexpression of an RNAi-insensitive version of HAAls, which contains a mutated oligo3_ORF target site (F and H). Expression of UAS-GFP or UAS-CG42306 did not rescue the alsRNAi phenotype (I and J). Expression of UAS-CG42306 did not cause a phenotype in the wing (K) and caused legs devoid of the entire tarsal region (L). (M) Ter94HA overexpression did not rescue the alsRNAi phenotype, and did not show any phenoype in the wing (N). (TIF) [file pbio.1001988.s002.tif]

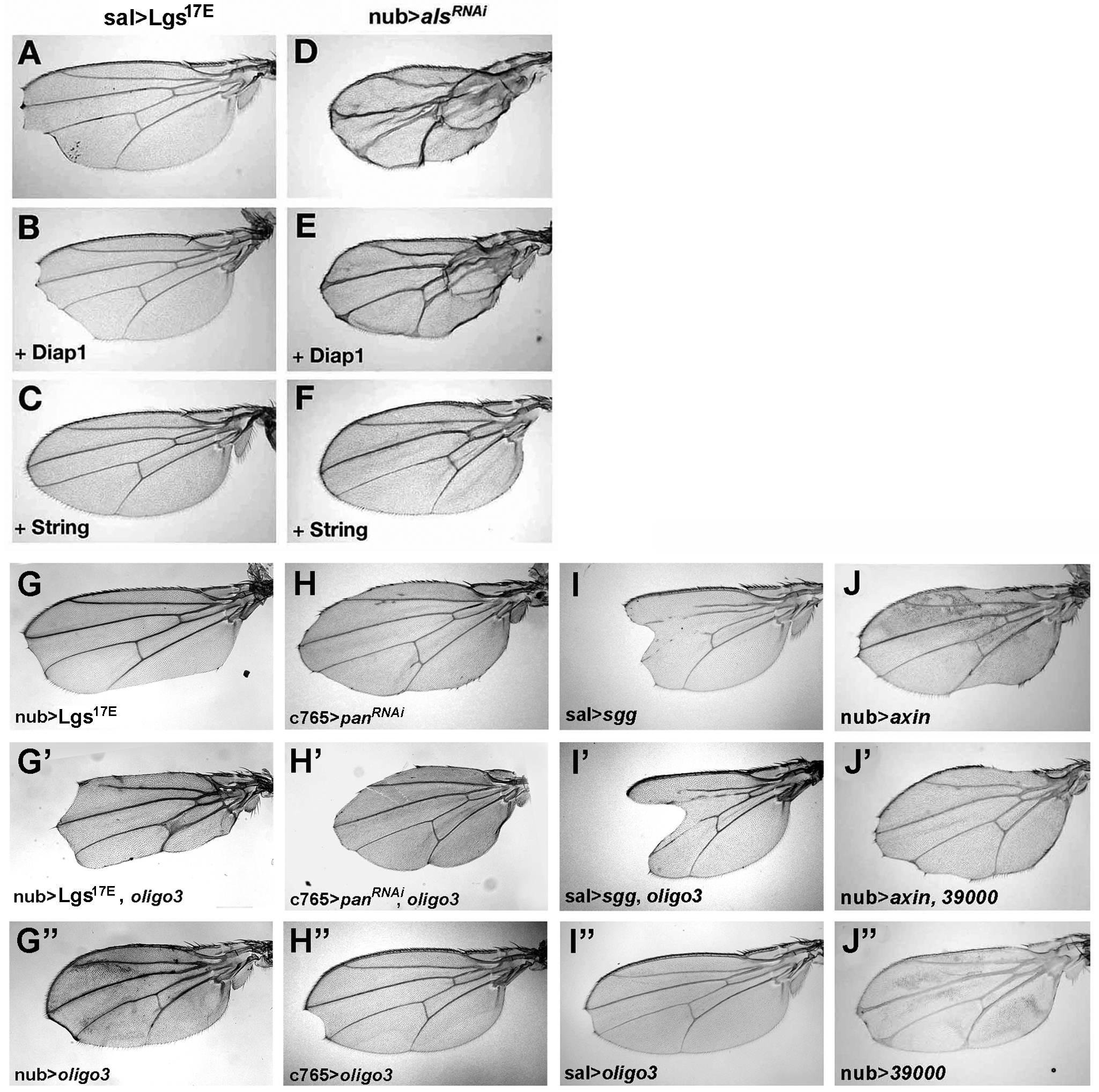

Supplement: Figure S3 — Genetic interaction of als with the cell cycle and Wg signaling. (A–F) Underproliferation, not apoptosis, underlies the alsRNAi wing phenotype. Overexpression of a negative Wg pathway component, Lgs17E (A), and of alsRNAi (D) causes wing margin notches. Inhibition of apoptosis by overexpression of Diap1 could not rescue the wing phenotypes (B and E), whereas acceleration of the cell cycle by overexpression of String could rescue the wing phenotypes (C and F). (G–J″) als interacts with the Wg pathway. Reduction of Wg signaling (G–J) was provoked by (G) overexpression of a dominant-negative mutant form of Legless, Lgs17E, (H) depletion of pangolin (pan), (I) overexpression of Shaggy (Sgg), or (J) overexpression of Axin. Simultaneously depleting als (G″–J″) leads to an enhancement of the notched wing margin phenotype observed with depleting als alone (G′–J′). (TIF) [file pbio.1001988.s003.tif]

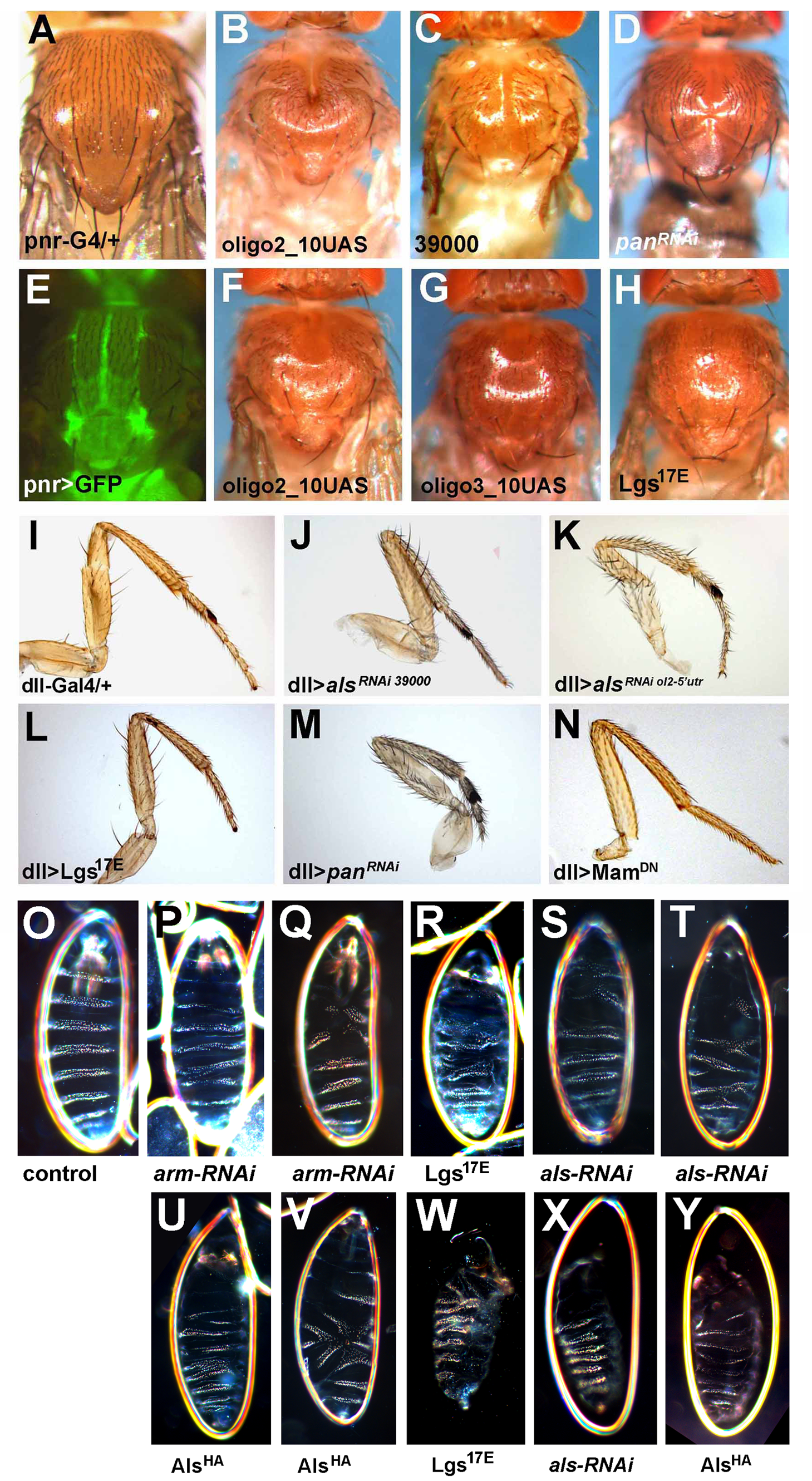

Supplement: Figure S4 — als depletion in the thorax and legs. (A–H) alsRNAi expression in the notum, the primordium of the thorax, by pannier-Gal4, which is active in the mesothorax (E). als depletion causes mild thoracic clefts (B and C), a slight polarity defect of thoracic bristles, reduced or lost dorsocentral microchaete, and an overall shortening of the thorax and scutellum (B, C, F, and G). These phenotypes are reminiscent of impaired Wg signaling, for example, based on panRNAi expression (D) or based on Lgs17E overexpression (H). (I–K) alsRNAi expression in the leg primordium by distalless-Gal4 caused, similarly to impaired Wg signaling, a shortening of tarsal segments and a dorsolateral shift of the sex combs (J, K, L, and M), along with a significant dorsalization (K and M). (N) Impaired Notch signaling caused a loss of segmental junctions along the tarsus, resulting in a complete fusion of the tarsal segments. (O–Y) Analysis of impaired als function in the embryo. Expression of alsRNAi or the dominant-negative-like AlsHA by da-Gal4 ([S] alsRNAi lines: oligo2_3′utr; [T] oligo310UAS) caused shorter cuticles than in control embryos, with ventral denticle belts partially fused (S–V, X, and Y). Expression of armRNAi (P and Q) or UAS-Lgs17E by da-Gal4 (R and W) caused similar phenotypes. (TIF) [file pbio.1001988.s004.tif]

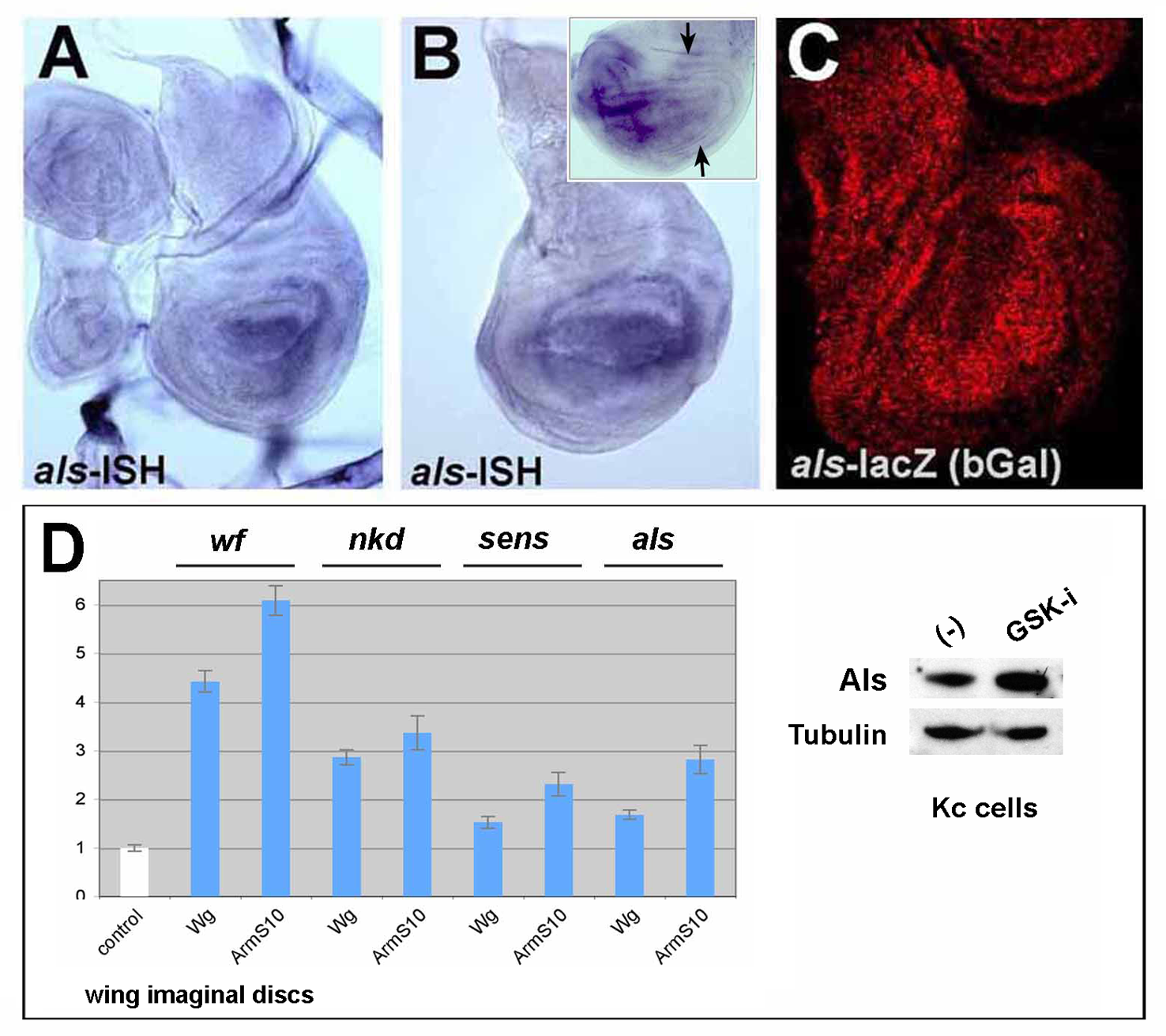

Supplement: Figure S5 — als is expressed in the center of the wing pouch. (A and B) als in situ hybridization reveals a pronounced expression of als in the center of the wing pouch and a low level of expression in the remaining part of the wing disc. (B) (inset) Expression of UAS-alsRNAi by hh-Gal4 in the P-compartment (arrows) of wing imaginal discs caused a strong decrease in als expression. (C) Reporter expression from a genomic als-lacZ transgene is similar to als expression visualized by in situ hybridization. (D) Activation of Wg signaling by the overexpression of either UAS-wg or UAS-armS10 with nubbin-Gal4 at 29°C in wing imaginal discs was monitored by the upregulation of the Wg targets wingful (wf), naked (nkd), and senseless (sens) (bars 2–7). Similarly, activation of Wg signaling caused an upregulation of als expression (bars 8 and 9). Als was upregulated upon stimulation of the Wg pathway by inhibiting GSK3β in Kc-167 cells (detected by anti-UBXN6 antibody). (TIF) [file pbio.1001988.s005.tif]

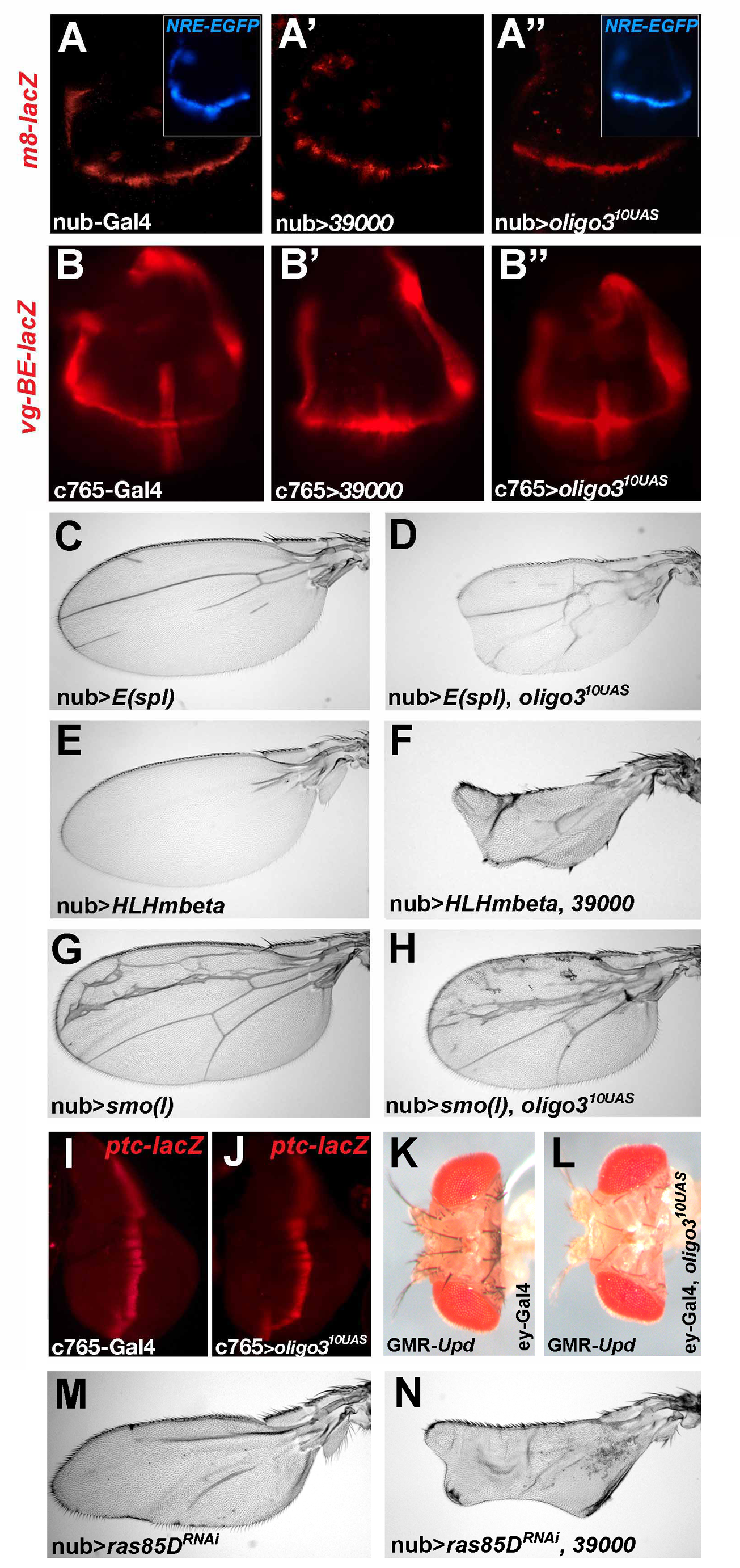

Supplement: Figure S6 — als does not interfere with Notch or Hedghog signaling. m8-lacZ expression at the D/V boundary of wing imaginal discs (A) is not altered upon als depletion (A′ and A″). NRE-GFP expression is not altered upon als depletion (inset of A and A″). vg-BE-lacZ expression (B) is not altered upon als depletion (B′ and B″). (C) Overexpression of E(spl), the nuclear mediator of Notch signaling, causes reduction and loss of wing veins. (D) E(spl) co-expressed with alsRNAi resulted in wings with lost veins combined with wing notches. (E) Overexpression of HLHmbeta, a positive target of Notch signaling, abolishes wing veins. (F) HLHmbeta co-expressed with alsRNAi resulted in veinless wings with marginal notches. (G) Overexpression of SmoI constitutively activates Hh signaling, which results in overgrown wings and ectopic veins. (H) Ectopic Hh signaling combined with alsRNAi resulted in a wing size reduction predominantly of distal tissue, without altering the phenotypes symptomatic for elevated Hh signaling, like overgrowth along the A/P axis or ectopic veins. (I and J) ptc-lacZ expression is not altered upon depletion of als. (K) Ectopic expression of Upd, the ligand of the Jak/Stat pathway, caused overgrown eyes, which was not altered upon co-expression of alsRNAi (L). (M) Impaired Ras/EGFR signaling caused slightly smaller wings lacking wing veins; co-expression of alsRNAi resulted in veinless wings with marginal notches (N). (TIF) [file pbio.1001988.s006.tif]

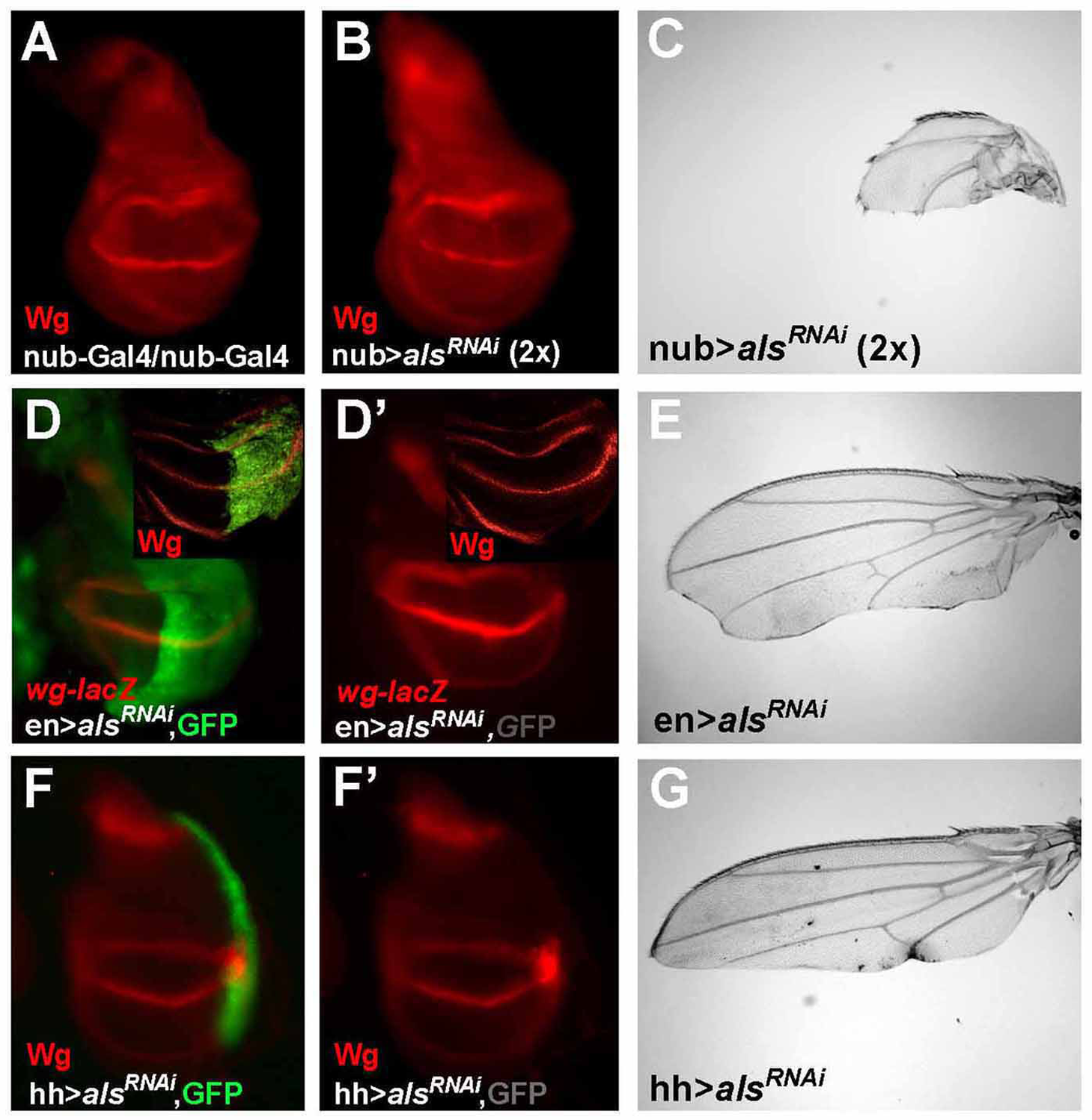

Supplement: Figure S7 — als depletion does not affect Wg expression. (A–F′) wg-lacZ and Wg protein expression is not altered upon expression of alsRNAi. Adult phenotypes corresponding to the respective genetic manipulations during wing disc development are shown for line 39000 (A–C), line oligo310UAS (D–E), and line oligo310UAS (F–G). (F–G) Upon strong RNAi expression by hh-Gal4 in the P-compartment (green, GFP expression), the P-compartment strongly undergrows, yet shows normal Wg expression. (TIF) [file pbio.1001988.s007.tif]

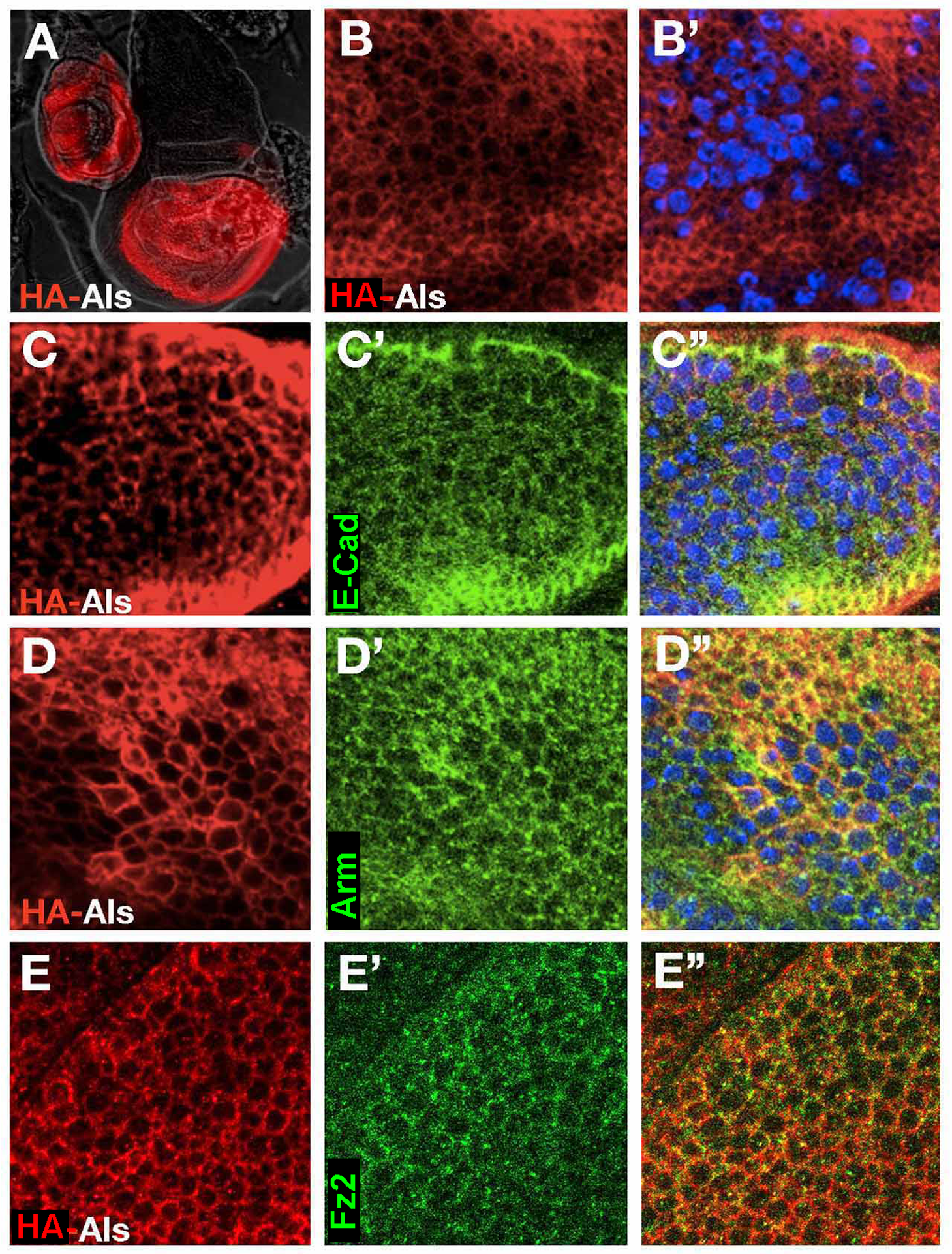

Supplement: Figure S8 — Als localizes adjacent to the apicolateral cell membrane. HAAls expressed with nubbin-Gal4 in the wing pouch localizes in the cytoplasm adjacent to the cell membrane (A–E″). HAAls localization overlaps to a large extent with that of E-Cadherin (C′ and C″), Arm (D′ and D″), and Fz2 (E′ and E″). Cell nuclei are blue (DAPI in the color-merged pictures [B′, C″, D″, and E″]). (TIF) [file pbio.1001988.s008.tif]

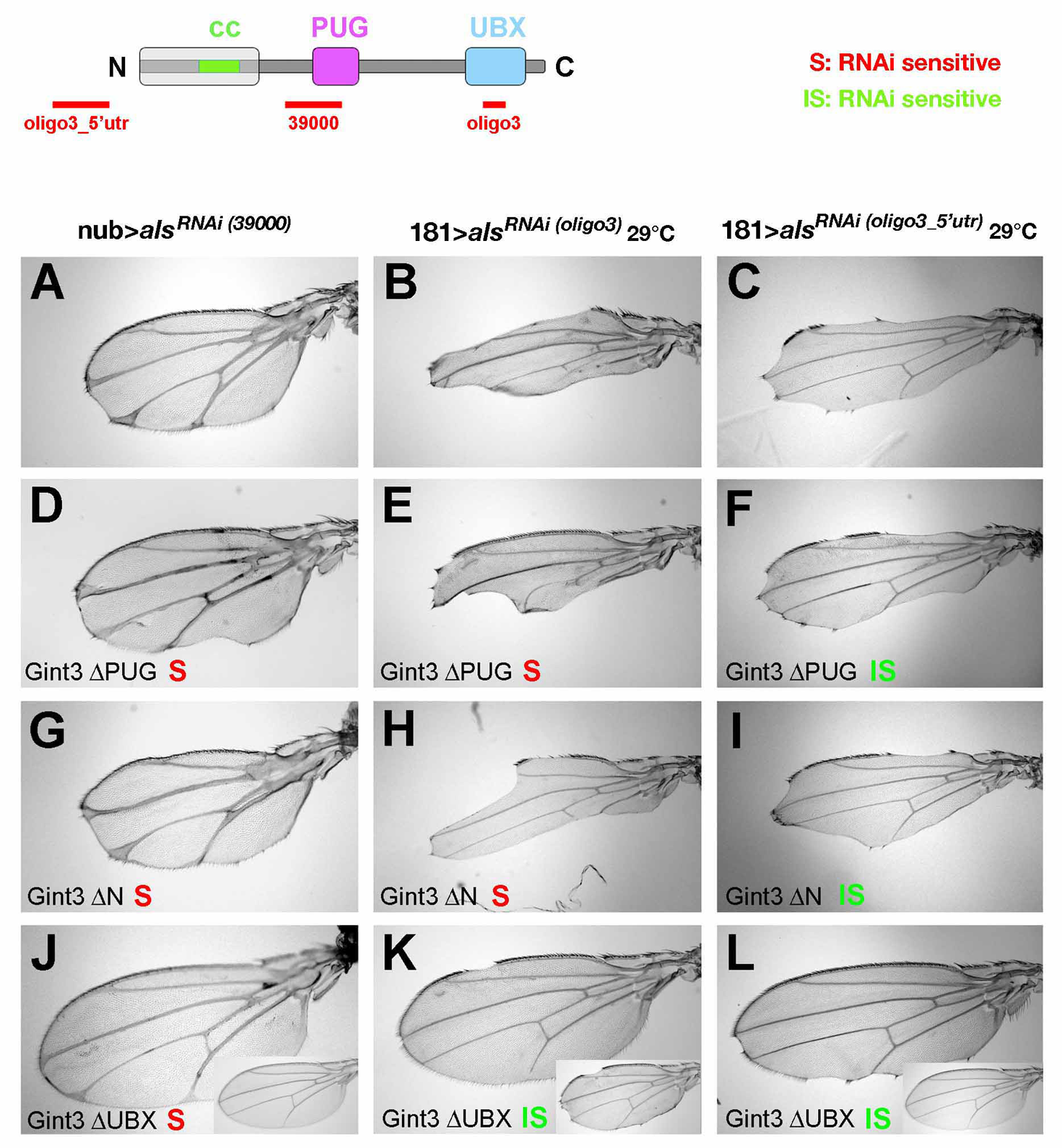

Supplement: Figure S9 — The PUG and the coiled-coil domains are essential for Als function. Expression of alsRNAi in the wing primordium with nubbin-Gal4 (A) or with 181-Gal4 (B and C) causes nicked wing margins. Co-expression of AlsΔPUG (D–F) or AlsΔN (G–I) did not alter the alsRNAi phenotype, whereas AlsΔUBX rescued the alsRNAi phenotype (J–L) comparably to full-length Als (insets of J–L). cc (coiled coil domain, within the N-terminal region of Als). (TIF) [file pbio.1001988.s009.tif]

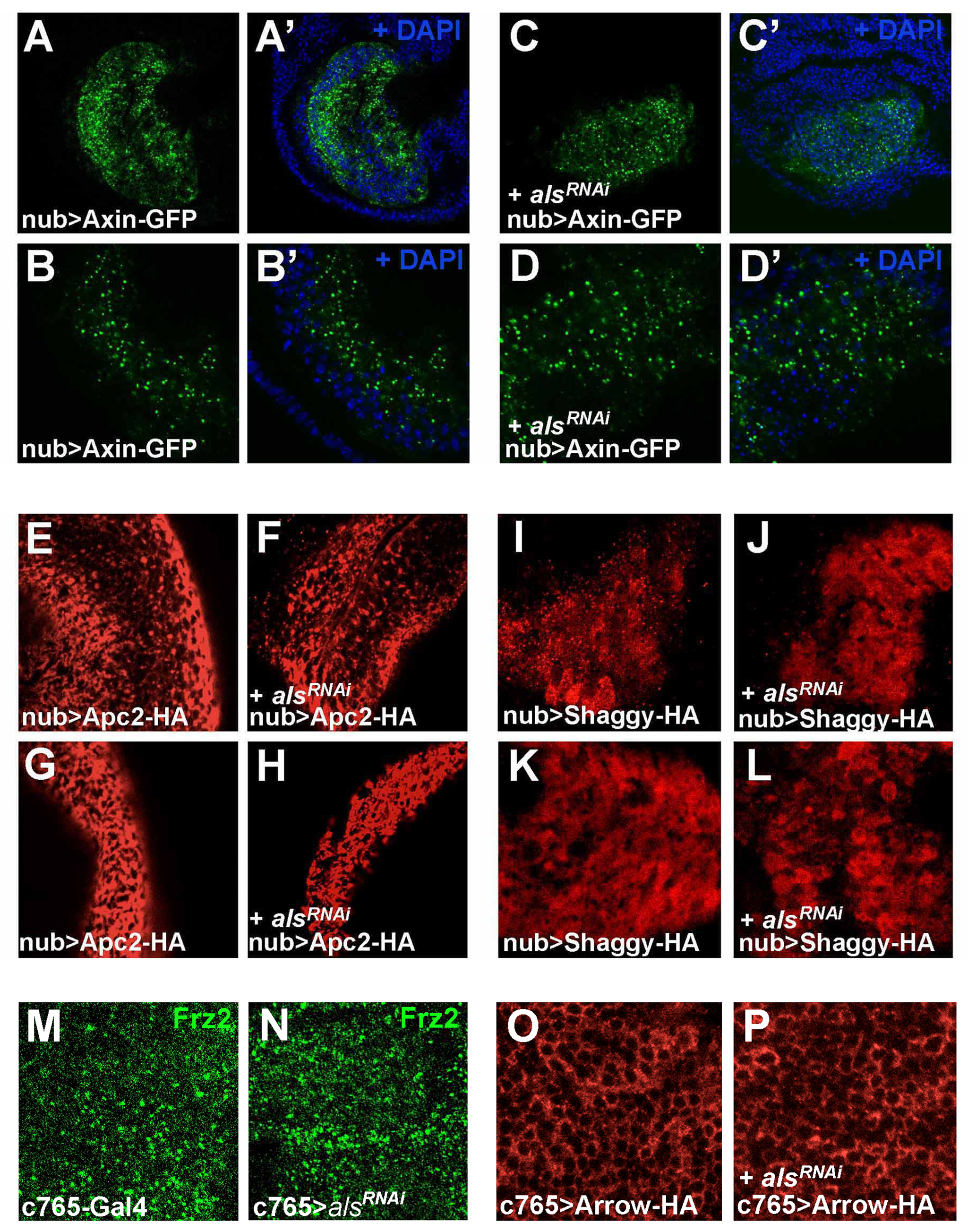

Supplement: Figure S10 — Negative pathway components are not altered upon als depletion. (A–D′) Axin-GFP expression levels are not changed upon depletion of als (line 39000 [C–D′, cf. A–B′]). Expression of Apc2HA (E–H) and ShaggyHA (I–L) is not altered upon als depletion (line 39000). (M–P) Expression levels of endogenous Fz2 (anti-Fz2-antibody staining) and ArrowHA were not altered upon depletion of als (line oligo310UAS, 29°C). Confocal pictures were taken at 40× magnification/2.5× zoom, except (A, A′, C, and C′): 40× magnification, 1× zoom; (E–L, O, and P): anti-HA antibody staining. (TIF) [file pbio.1001988.s010.tif]

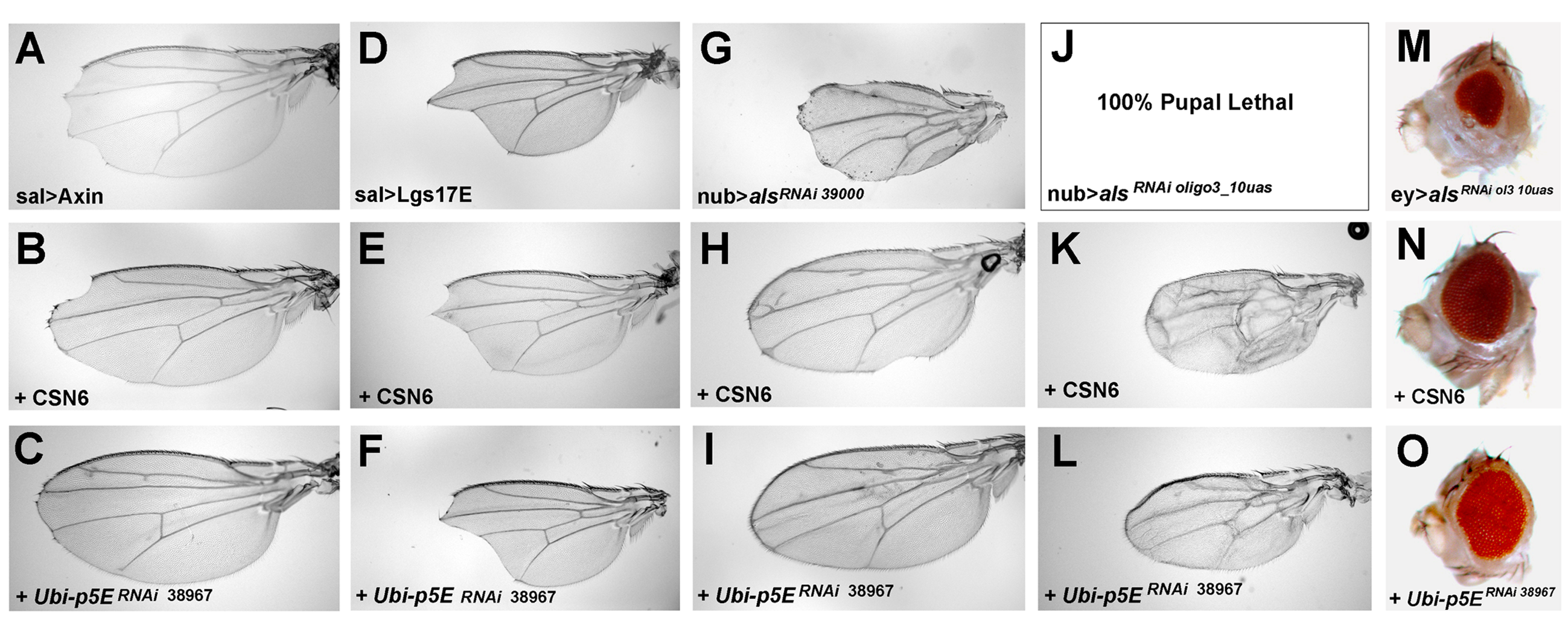

Supplement: Figure S11 — Als acts upstream of Arm's proteasomal degradation. The overexpression of CSN6 suppressed als RNAi phenotypes (H, K, and N, cf. G, J, and M) as well as a Wg loss-of-function phenotype caused by Axin overexpression (B, cf. A), but not as well as a Wg loss-of-function phenotype caused by the overexpression of Lgs17E (E, cf. D). Similarly, depletion of ubiquitin could suppress phenotypes based on als RNAi (I, L, and O, cf. G, J, and M) and Axin overexpression (C, cf. A), but could not ameliorate the Lgs17E overexpression phenotype (F, cf. D). (TIF) [file pbio.1001988.s011.tif]
