# Supplementary material for: Protection of Armadillo/β-Catenin by Armless, a Novel Positive Regulator of Wingless Signaling
Source: PLoS Biol. 2014 Nov 4;12(11):e1001988. doi: 10.1371/journal.pbio.1001988 (PMC4219662; doi:10.1371/journal.pbio.1001988)
Supplement: Text S1 — Supporting information. (DOCX) [file pbio.1001988.s012.docx]

**Supporting Information - Text S1**

**Nomenclature of Armless**

*CG5469* is annotated in Flybase as *Gint3* based on a conference abstract [1] where Gint3 was found to interact with Rab-GDI/*CG4422* and was therefore named “GDI interacting protein 3”. However, no data has ever been published. Furthermore, in several, independent pull-down experiments followed by mass spectroscopic analyses we could not find evidence that *CG5469* interacts with *CG4422*. We refer to *CG5469* as *armless (als)* throughout our work.

***armless (als)* acts in a dominant-negative like manner**

Expression of wild-type Als (*UAS-als*) was mostly phenotypically neutral; however, stronger expression resulted in phenotypes identical to *als* depletion, i.e. wing notches (Figure S2A). We attribute this to a dominant-negative (DN) behaviour of Als upon overexpression. The DN-like phenotype became even more apparent upon overexpression of Als^HA^, a C-terminally epitope tagged version (Figure S2B).

**Rescue of *als* RNAi phenotypes by ^HA^Als**

Because of its DN-like phenotype (Figure S2A inset and Figure S2B), overexpression of Als could only partially rescue the *als^RNAi^* phenotype (Figure S2F). The DN-like-effect was weakest when Als was N-terminally tagged (^HA^Als), a form which we then used for *als* RNAi rescue experiments. We applied two rescue strategies: (i) We used *als^RNAi^* lines targeting the 5’ UTR of endogenous *als* (Figure S2C), which could be rescued upon overexpression of an *UAS-^HA^als* transgene, lacking endogenous UTRs (Figure S2D). (ii) We used the *als^oligo3_10UAS^* RNAi line which targets the coding region of endogenous *als* (Figure S2E,G) in combination with an RNAi-insensitive rescue version of ^HA^Als (see Materials and Methods). The *oligo3^RNAi^* phenotype could largely be rescued (Figure S2F,H). To test whether an additional *UAS*-transgene could *per* *se* titrate the available Gal4, and thus suppressing the *als^RNAi^* phenotype, we co-overexpressed unrelated *UAS*-transgenes (*UAS-GFP* or *UAS-CG42306*, see next paragraph) together in *Gal4*>*als^RNAi^* backgrounds. Co-overexpression of these transgenes did not alter or rescue the *als^RNAi^* phenotype (Figure S2I,J).

**The dicistronic genomic locus of *als* and *CG42306* can be functionally separated**

In *Drosophila*, approximately 40% of the transcripts contain upstream start codons (uAUGs) with very short ORFs but there is little evidence that these are translated and affect their associated mORF [2]. The open reading frame (ORF) of *als* is preceded by an upstream ORF (*CG42306*), which encodes a short peptide with a transmembrane domain, while the 3’-UTR of *CG42306* overlaps with the coding sequence of *als*, and the 5’UTR of *CG42306* overlaps partially with the 5’UTR of *als*. Therefore, RNAi target sites used for *als* depletion are also complementary to *CG42306*. Furthermore, in two other *Drosophila* species the *CG42306*-uORF is fused to the ORF of Als by transcript splicing, resulting in a Als variant which contains an N-terminal transmembrane domain. Although annotated as two separate ORFs in FlyBase we wanted to confirm that a *CG42306*-*als* fusion does not exist and that *CG42306* does not underlie the *als^RNAi^* phenotypes described in our study:

(i) Sequence analysis of cDNA (based on total RNA isolation of L3 wing discs) by independent, various PCRs revealed that there is no translational frame maintained between *CG42306* and *als* (5 bp separate the STOP codon of *CG42306* and the START-codon of *als*). It is therefore unlikely that a CG42306-Als fusion protein exists as shown for *Drosophila* *pseudoobscura* and *grimshawi* (STRING database). (ii) The *als^RNAi^* phenotypes of the wing or the eye was not rescued, and not altered at all, upon overexpression of the putative coding region of *CG42306* (*UAS-CG42306*; Figure S2J,K). (iii) Overexpression of *CG42306* did not interact with Wg signalling. It did cause a leg phenotype that is reminiscent of a strong EGFR signalling gain-of-function phentoype (Figure S2L); this phenotype differs completely from any Wg-related scenarios. (iv) The *als^RNAi^* phenotype was rescued by *UAS-als* and *UAS-UBXN6*, the human ortholog of Als. *CG42306* lacks a human ortholog, and is only found in fly species. (vi) No *CG42306*-encoded protein was so far identified (Protein Peptide Atlas). (vii) In case of a protein fusion the transmembrane domain, encoded by *CG42306*, would lie N-terminally. Consequently, the entire Als protein part would lie extracellularly. This would not be in agreement with our observed Als-Ter94 protein interaction, since Ter94 is a cytoplasmic protein, nor with the annotated cytoplasmic functions of the PUG- and UBX-domain, nor with our epistatic analysis where we found Als to function downstream of cytoplasmic components of Wg signalling.

**Mass spectrometry results**

In total, we performed five independent immunopurification experiments in Drosophila Kc-167 cells overexpressing different HA-tagged variants of the Als protein (for details see Materials and Methods). The affinity-purified protein samples were digested into peptides and subjected to shotgun LC-MS/MS in an orbitrap mass spectrometer. Consistently, in all experiments we could detect Ter94 next to the Als bait protein top ranking with a high protein sequence coverage (between 65-80%). Other potential interactors not identified in the control sample include cytoskeleton-associated proteins. A representative result for one transfection experiment (overexpression of N-terminal tagged ^HA^Als in Kc-167 cells) is given below. Detected peptides are shown in bold.

Armless/Als (FBpp0085836); protein score 9174; sequence coverage 85% (ranked hit 2):

| 1 | MSKIKKFFSR | **KKAEAAFKLN** | **LTGSGMGTGH** | **K**LNSPK**QETP** | **SSSSRQKYEA** |
| --- | --- | --- | --- | --- | --- |
| 51 | **YVPPKRNEIS** | **NEARAAASAA** | **LAR**IDKKTSR | **EFNTSLSAVK** | AQAK**RELEAE** |
| 101 | **RRQREEAMGT** | **PSTTSTSTSA** | **SGGDTRNLAC** | **EGVFFRCPLI** | **SEEILPK**SVW |
| 151 | KVR**IKEFLYQ** | **QLEADRGLTA** | **CLIIHNCNVK** | **EKADECIATL** | **IRYLENLIKN** |
| 201 | **PEEEKFCK**IR | MSNKIFSEK**V** | **RYVEGALDVL** | **QAAGFNEVQI** | **DGEPFLLWTK** |
| 251 | **EQTEKDLDLP** | **TLVEALKSSE** | **IIPLELDR**NI | K**VLLPSQACR** | **VALPDEFYRL** |
| 301 | **SPEEIKKEQQ** | **LRSEAIAQSQ** | **MLR**TKAMR**ER** | **EEQR**NLRMYR | YALVR**VKFPN** |
| 351 | **GLFIQGTFNV** | **YEKISDVFEF** | **VQSCLADESL** | **DFSLVSNSDG** | **KLGDEDLEKT** |
| 401 | **LYDCKLIPNT** | **LLLFSANDTP** | **APLQTDINYL** | **KEDLLMLVQA** | **M** |

Ter94 (FBpp0087479); protein score 5927; sequence coverage 65 % (ranked hit 3):

| 1 | M**ADSKGEDLA** | **TAILK**RKDRP | NR**LIVEEAQN** | **DDNSVVSLSQ** | **AKMDELQLFR** |
| --- | --- | --- | --- | --- | --- |
| 51 | **GDTVILK**GKR | RK**ETVCIVLS** | **DDTCPDEK**IR | MNRVVRNNLC | VHLSDVVSVQ |
| 101 | SCPDVKYGKR | VR**ILPIDEST** | **EGVTGNLFEI** | **YLKPYFLEAY** | **RPIHMGDNFI** |
| 151 | **VR**AAMRPIEF | K**VVLTDPEPY** | **CIVAPETVIF** | **CDGDPIKREE** | **EEESLNAVGY** |
| 201 | **DDIGGCRK**QL | AQIK**EMVELP** | **LRHPSLFKAI** | **GVKPPRGILM** | **YGPPGTGK**TL |
| 251 | IAR**AVANETG** | **AFFFLINGPE** | **IMSKLAGESE** | **SNLR**KAFEEA | EK**NSPAIIFI** |
| 301 | **DEIDAIAPK**R | DK**THGEVER**R | **IVSQLLTLMD** | **GMKK**SSHLIV | MAATNRPNSI |
| 351 | DPALRRFGRF | DR**EIDIGIPD** | **ATGR**LEVLRI | HTKNMKLHDD | VDLEQIAAES |
| 401 | HGHVGADLAS | LCSEAALQQI | R**EKMDLIDLE** | **DDKIDAEVLA** | **SLAVTMENFR** |
| 451 | YAMTKSSPSA | LR**ETVVEVPN** | **TTWTDIGGLE** | **SVKKELQELV** | **QYPVEHPDK**F |
| 501 | LK**FGMQPSRG** | **VLFYGPPGCG** | **K**TLLAK**AIAN** | **ECQANFISVK** | **GPELLTMWFG** |
| 551 | **ESEANVR**DIF | DKAR**SAAPCV** | **LFFDELDSIA** | **K**AR**GGNVGDA** | **GGAADRVINQ** |
| 601 | **ILTEMDGMGA** | **K**KNVFIIGAT | NRPDIIDPAI | LRPGR**LDQLI** | **YIPLPDDK**SR |
| 651 | EAILKANLRK | SPLAK**EVDLT** | **YIAKVTQGFS** | **GADLTEICQR** | ACKLAIR**QAI** |
| 701 | **EAEIR**REKER | **AENQNSAMDM** | **DEDDPVPEIT** | **SAHFEEAMK**F | ARRSVSDNDI |
| 751 | R**KYEMFAQTL** | **QQSRGFGQNF** | **R**FPGQTGNTS | GSGNNLPVNS | PGDNGDDDLY |
| 801 | S |  |  |  |  |

p47 (FBpp0088069), Ter94-HA complex, sequence coverage 7%, 1 peptide:

| **1** | MAARGDLIAQ | FIEITGTDEN | VARFYLSSCD | WDIEHALGNY | WSTQADLPVP |
| --- | --- | --- | --- | --- | --- |
| **51** | VPTVGHADNP | KPKPTSSSGA | SASASAAGAT | KSADSAVATS | SASVDIAPAA |
| **101** | TKAKPKFATL | SDMSKESSSD | DDQQAFYAGG | SDRSGQQVLG | PPKRKNFREQ |
| **151** | LTDMMRSAQE | QNIAEVGPST | SSGSASGGSG | GAVWGQGMRL | GMTDNDHTAV |
| **201** | GTKKPAATIE | NKPVVVLKLW | SQGFSIDGGE | LRHYDDPQNK | EFLETVMRGE |
| **251** | IPQELLEMGR | MVNVDVEDHR | HEDFKRQPVP | QTFKGSGQK**L** | **GSPVANLVTE** |
| **301** | **APTVPVALSP** | **GEAANQEASA** | **R**DAINLNSEA | PSTTLQIRLA | DGSRLAAQFN |
| **351** | LSHTVSDIRR | FIQTARPQYS | TSNFILVSSF | PTRELSDDNS | TIEKAGLKNA |
| **401** | ALMQRLK |  |  |  |  |

p47 (FBpp0088069), UAS-Als-HA complex, sequence coverage 21%, 5 peptides:

| **1** | MAARGDLIAQ | FIEITGTDEN | VARFYLSSCD | WDIEHALGNY | WSTQADLPVP |
| --- | --- | --- | --- | --- | --- |
| **51** | VPTVGHADNP | KPKPTSSSGA | SASASAAGAT | K**SADSAVATS** | **SASVDIAPAA** |
| **101** | **TK**AKPK**FATL** | **SDMSK**ESSSD | DDQQAFYAGG | SDR**SGQQVLG** | **PPK**RKNFREQ |
| **151** | LTDMMRSAQE | QNIAEVGPST | SSGSASGGSG | GAVWGQGMRL | GMTDNDHTAV |
| **201** | GTKKPAATIE | NKPVVVLKLW | SQGFSIDGGE | LRHYDDPQNK | EFLETVMRGE |
| **251** | IPQELLEMGR | MVNVDVEDHR | HEDFKRQPVP | QTFKGSGQK**L** | **GSPVANLVTE** |
| **301** | **APTVPVALSP** | **GEAANQEASA** | **RDAINLNSEA** | **PSTTLQIR**LA | DGSRLAAQFN |
| **351** | LSHTVSDIRR | FIQTARPQYS | TSNFILVSSF | PTRELSDDNS | TIEKAGLKNA |
| **401** | ALMQRLK |  |  |  |  |

In shotgun affinity purification mass spectrometry using Arm or Arm^S10^ as a bait established components of the degradation complex like Apc2, Axin, CK1 and Sgg were detected in the cytoplasm (detected peptides are listed below):

| **CG6193** | **Apc2** | IIENAGGILR |
| --- | --- | --- |
|  |  | TFEIDLDQPTDFSAR |
|  |  | TIFNELGTVTSLAR |
|  |  |  |
| **CG7926** | **Axin** | ETNTFQVIPR |
|  |  | NPGAALLTNER |
|  |  | APDYPIR |
|  |  | THCEDPDSPVIQEEIVNDSDILPLFGDK |
|  |  |  |
| **CG2028** | **CKIalpha** | ILSGGVGFPR |
|  |  |  |
| **CG2621** | **Sgg** | APELIFGAINYTTK |
|  |  | ITTVVATPGQGTDR |

**References for Text S1**

1. [Cheney C, Amin P, Cope A, Lawson M, Richardson B *et. al.*](http://flybase.org/cgi-bin/fbidq.html?FBrf0198360) (2007) Interaction of *Drosophila* rab GDI with Gint3, a ubiquitin-domain-containing protein. *A. Dros. Res. Conf.* 48: 278B.

2. Hayden CA, Bosco G (2008) Comparative genomic analysis of novel conserved peptide

upstream open reading frames in *Drosophila melanogaster* and other dipteran species. BMC Genomics 9: 61-74.
